# Nitrogen-doped ZnO and TiO_2_ supported on activated carbon for dual pollutant degradation under UV/H_2_O_2_ process

**DOI:** 10.1038/s41598-025-32739-8

**Published:** 2026-01-22

**Authors:** H. Mandour, O. Abdelwahab, N. K. Amin, E.-S. Z. El-Ashtoukhy

**Affiliations:** 1 Chemical Engineering Department, Faculty of Engineering, Alexandria University, Alexandria, Egypt; 2 National Institute of Oceanography and Fisheries, NIOF, Alexandria, Egypt

**Keywords:** Nitrogen-doped, Granular activated carbon (GAC), Photo-degradation, Ammonia, Phenol, RSM, Chemistry, Engineering, Environmental sciences, Materials science, Nanoscience and technology

## Abstract

Nitrogen-doped ZnO and TiO_2_ nanoparticles supported on GAC were simulated and characterized. Various techniques including SEM, EDX, XPS, Raman, and DRS were employed to confirm both the successful nitrogen doping and the effective immobilization of ZnO and TiO₂ particles onto the GAC substrate. The resulting N-ZnO/AC and N-TiO_2_/AC catalysts were applied in the photo-degradation of ammonia and phenol within a semi-continuous flow photocatalytic reactor. Photocatalytic activity assessments were performed on both catalysts with flow rate and pH variations. These investigations indicated that the optimal degradation of both contaminants occurred at 8 L/min flowrate and a moderate pH level. To comprehensively evaluate the impact of various independent parameters on degradation efficiency, Response Surface Methodology (RSM) was applied. Optimization of the UV/Catalyst/H_2_O_2_ process for the N-ZnO/AC catalyst was conducted using a Box-Behnken design. The predicted photo-degradation efficiency for both ammonia and phenol were found in excellent agreement. Optimization process revealed that the maximum photo-degradation efficiency was achieved under specific conditions: 120 min of irradiation time, 0.86 g L^− 1^ catalyst dose, an H_2_O_2_ concentration of 45 mM, an initial ammonia concentration of 96.55 ppm, and an initial phenol concentration of 10 ppm.

## Introduction

Industrialization has led to the release of diverse chemical wastes into surrounding ecosystems, making it a major contributor to water pollution^1,2^. These wastes include heavy metals, sludge, solvents, and toxic organic and inorganic compounds. Among them, ammonia nitrogen and phenol are particularly concerning due to their significant impacts on both human health and ecological systems^1,3^.

Phenol is the most common aromatic compound which usually recognized as serious environmental pollutants even though it presents in a few concentrations in water it would be highly carcinogenic for human health “allowable concentrations as per US Environmental Protection Agency (USEPA) regulations ˂ 1 ppm”^2,3^. Phenol not highly bio-accumulative, its degradation products may pose additional risks. In humans, phenol exposure can result in skin burns, systemic toxicity, and respiratory irritation, while chronic exposure may affect liver, kidney, and nervous system function. The OSHA PEL for phenol is 5 ppm, and it is classified by the International Agency for Research on Cancer (IARC) as Group 3, indicating it is not classifiable as to its carcinogenicity to humans Ammonia causes significant effects on ecosystem and human health. Ammonia contributes to air pollution through the formation of fine particulate matter (PM2.5), and its release into aquatic systems can lead to eutrophication, oxygen depletion, and aquatic life mortality. It also promotes soil acidification, disrupting microbial balance and reducing fertility. It affects the respiratory system, skin, and eyes, and can cause death at concentrations exceeding 300 ppm^4,5^.

The wastewater treatment is still commonly performed using a variety of conventional techniques as physical, chemical, biological processes. However, these processes have some drawbacks as production of secondary contaminants and how to get ride or dispose of these contaminants, limitation of application, high cost for capital and operating cost for the process^6–8^.

Accordingly based on these reasons, Advanced Oxidation Processes (AOPs) have evidently considered as a promising alternative techniques instead of wastewater conventional methods as AOPs significantly reduce the operational cost by operating at ambient temperature and pressure^9^.

AOPs generate strong oxidizing free radicals as ·OH, ·O_2_^–^ and HO_2_· which actively attack and oxidize contaminants such as NO_X_, organic compounds, H_2_S and trace elements^10^. Among these, UV/H_2_O_2_ technology has gained a lot of consideration as a representative of AOPs for the treatment of water. This process enhances and accelerates pollutant oxidation by generating additional ·OH and ·O_2_^–^ radicals through the photolysis of H₂O₂^10,11^.

Titanium dioxide (TiO_2_) and zinc oxide (ZnO) are the most commonly used photocatalysts due to their chemical stability, strong oxidative potential, low cost, and effective photocatalytic activity. However, their powder forms present challenges, both TiO₂ (Eg = 3.3 eV) and ZnO (Eg = 3.2 eV) require high energy input to generate ·OH radicals, suffer from high electron-hole recombination rates, and tend to agglomerate in solution, which complicates separation and reduces photocatalytic efficiency^2,12–15^.

Immobilization of TiO_2_ and ZnO powder catalyst on a proper, floating, and inert support can enhance catalyst separation process as as zeolite, glass, fly ash, perlite granules and activated carbon^16,17^.

In this work, bi-functional composites have been developed by nitrogen-doped photo-catalyst immobilized on activated carbon (N-Doped TiO_2_ /AC and N-doped ZnO / AC). Choosing nitrogen as a dopant and granular activated carbon (GAC) as a carrier for photo-catalyst due to the following reasons: (a) nitrogen doping reduces photo-generated electrons and holes pairs recombination rate and narrowing band gab energy by supplying additional electrons^18,19^ and (b) GAC can be utilized as a superior and cost- effective adsorbent for a removal of organic pollutants and has been extensively applied in wastewater treatment processes^20–22^.

Response Surface Methodology (RSM) has proven to be a powerful tool for optimizing photocatalytic processes. Among RSM designs, the Box-Behnken Design (BBD) is particularly favored for its efficiency and reliability in evaluating process parameters^16,23,24^.

The current work was under-taken to assess the phenol and ammonia degradation by UV/photo-catalyst/H2O2 process with a synthesized N-doped TiO2 /AC and N-doped ZnO /AC catalysts using semi-continuous photo-reactor and study the effect of five independent variables (Irradiation Time (min), Catalyst Dose (g/L), H2O2 Concentration (mM), Ammonia Initial Concentration (ppm) and Phenol Initial Concentration (ppm)) using RSM.

## Materials and methods

### Materials

Commercial Granular Activated Carbon (GAC-Merck), Hydrochloric acid (30%) ,Titanium Dioxide (Sigma-Aldrich) with purity of at least 99.5%, Zinc nitrate hexahydrate (Zn (NO_3_)_2_·6H_2_O, Sigma-Aldrich), Titanium(IV) Isopropoxide (TIP) (Sigma-Aldrich), Ammonia (30–35%), Acetic acid (Merck,99.8%), Ethanol 99%, Urea (Merck),, Sodium Hydroxide, Zinc Oxide (ZnO_2_, Sigma-Aldrich), Phenol (Merck), Ammonium Chloride (Merck), Deionized Water (Sigma-Aldrich), Distilled Water.

### Preparation of photo-catalyst

N-doped ZnO- AC / N-doped TiO_2_ -AC catalyst (N-ZnO/AC and N-TiO_2_/AC) were produced via a modified sol- gel technique which was based on GAC as follows^25^: Nine grams of GAC were mixed with 5% acetic acid (300 ml) for 1 h to form an acetic acid solution. Eighteen gram of TIP were dissolved in 20 ml of hydrochloric acid (36%) and then mixed water (100 ml) to form a titanium chloride solution. One gram of TiO_2_ was added to 3.0 g of urea and 60 mL of ethanol, and the mixture was stirred until the solvent evaporated to form the “N-Doped Solution”. The N-doped solution and titanium chloride solution were added to the GAC solution and mixed for 3 hours. The Ti-activated carbon solution was then added dropwise to 50% ammonia solution (400 ml) with consistent mixing. The resulting gel – like macrospheres were stabilized in the ammonia solution for 1 h, washed twice with ethanol, washed five times with distilled water, dried, and calcined at 400 °C to obtain the N-TiO_2_/AC catalyst.

**Fig. 1:**
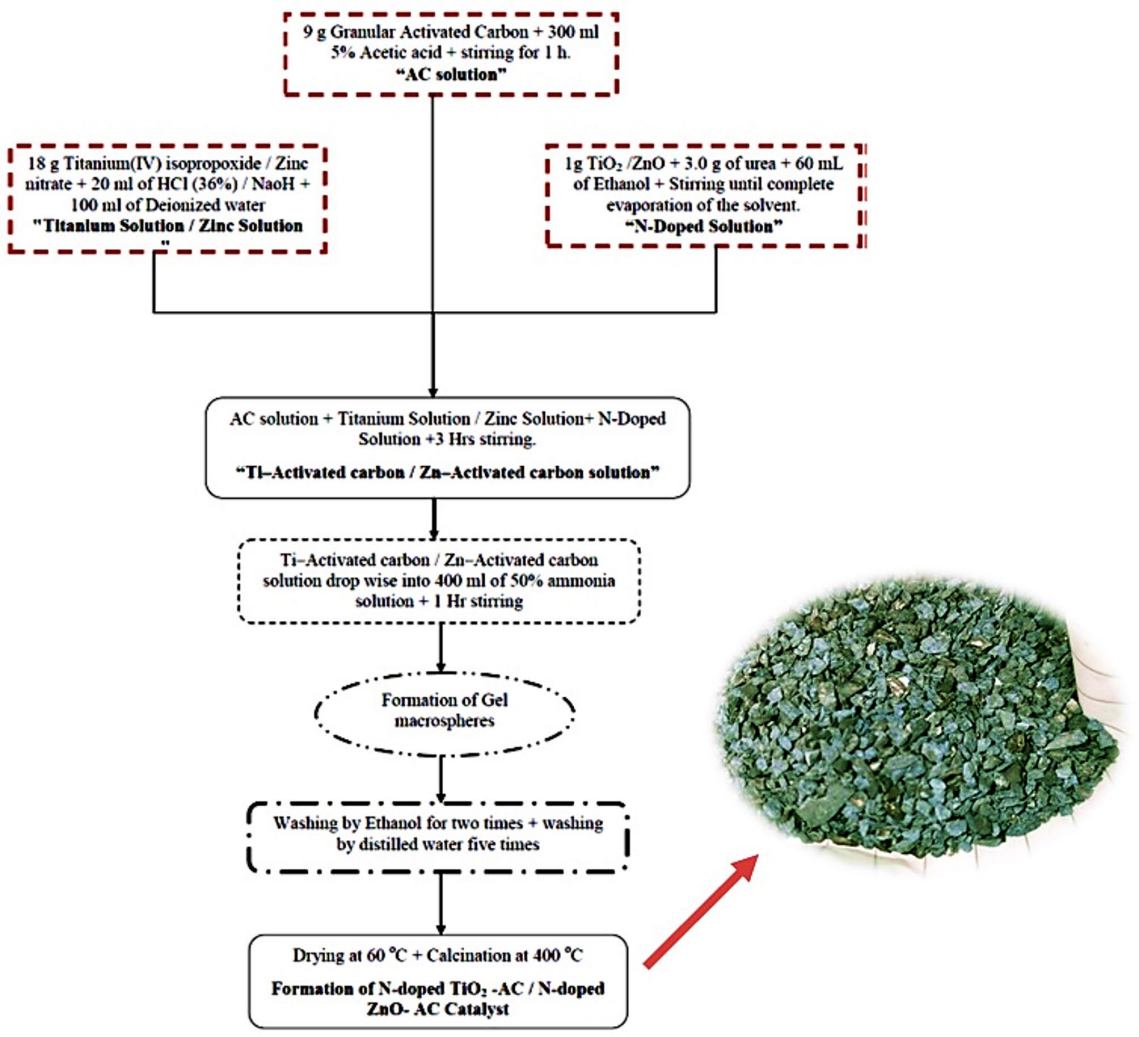
Procedure of N-doped TiO_2_ -AC / N-doped ZnO- AC catalyst preparation.

The synthesis of the N-doped ZnO-AC catalyst followed the same procedure as the N-doped TiO2-AC catalyst to obtain the N-ZnO/AC catalyst as shown in Fig. 1.

### Characterization of photo-catalysts

SEM (Scanning Electron Microscopy) was employed to analyze the materials’ elemental composition and surface morphology using a JSM-IT200 In Touch Scope equipped with a Fully integrated energy-dispersive X-ray spectroscopy (EDS) system featuring live analysis capabilities (JEOL, Akishima, Tokyo).

X-ray Photoelectron Spectroscopy (XPS) measurements were conducted using a K-Alpha spectrometer (Thermo Fisher Scientific, USA) with monochromatic Al Kα radiation. The analysis was performed under ultra-high vacuum conditions (~ 10^–9^ mbar), utilizing a full-spectrum pass energy of 200 eV and a narrow-spectrum energy of 50 eV. The binding energy range scanned extended from − 10 to 1350 eV.

Raman spectroscopy was carried out using a SENTERRA Raman microscope (Bruker) with a 785 nm excitation wavelength to investigate the crystalline phases of the catalysts. All measurements were performed with a scan range of 0–1000 cm^–1^ and an exposure time of 3 s per run.

To evaluate the optical properties of the materials, Diffuse Reflectance Spectroscopy (DRS) was performed using a JASCO V-570 UV–Vis spectrophotometer, covering a wavelength range from 250 to 850 nm.

### Photo-degradation trials

**Fig. 2:**
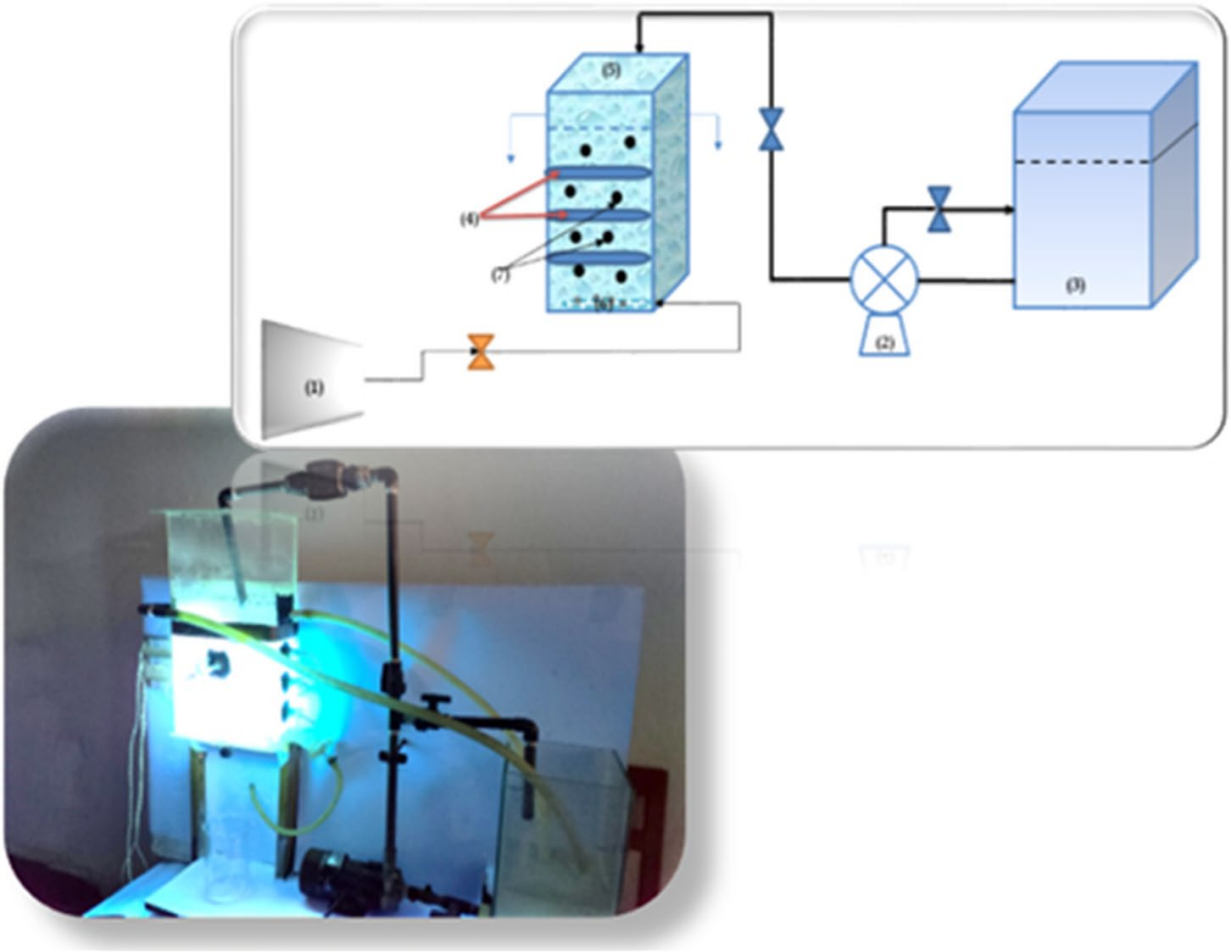
Photocatalytic reactor system : 1—air compressor, 2—circulation pump, 3—feed tank, 4–UV lamps, 5—reactor, 6–air bubbles, 7–catalyst.

Our Trials were conducted in a semi-continuous flow photocatalytic reactor as described in our earlier work^26^ as shown in Fig. 2. Degradation experiments were performed using a binary aqueous solution containing ammonia (300 mg/L) and phenol (100 mg/L) with a total volume of 8 L in the feed tank. The solution was continuously mixed in the dark for 30 min prior to the photo-reaction to reach adsorption-desorption equilibrium. The reactor was then charged with 10 mM H_2_O_2_ and 8 g of catalyst per 8 L of tank volume. After 180 min of experimentation, samples were taken at intervals of 5, 10, 15, 30, 60, 90, 150, and 180 min, and filtered through a 0.2 μm filter paper to remove any small catalyst particles. The circulation flow rate was varied between 0.25 and 8 L/min using a digital flow meter (Professional K24 flow meter - China). The pH values were adjusted using 1 N NaOH and HCl solutions, and pH was adjusted using a pH meter (HANNA, Japan). The point of zero charge for both N-TiO_2_/AC and N-ZnO/AC was determined using the potassium nitrate method^27^. The pollutant degradation efficiency was calculated using the following Eq. (1):

1 $${\mathrm{~Degradation}}={\mathrm{~}}\left[ {\frac{{{{\mathrm{C}}_{\mathrm{o}}} - {\mathrm{~C}}}}{{{{\mathrm{C}}_{\mathrm{o}}}}}} \right]{\mathrm{~}} \times 100{\mathrm{~}}$$

Where C_0_ represents the initial equilibrium concentration of the contaminants, and C denotes the final concentration after UV illumination. The concentration of ammonia nitrogen was measured using the ASTM Standard Method 4500, specifically the Phenate Method with spectrophotometric detection. The concentration of phenol was determined according to ASTM Standard Method 5530, employing the 4-Aminoantipyrine Method with spectrophotometric analysis.

### Experimental design and optimization

Experimental design is a method to assess the value of factors, their interactions, and manipulate them to obtain optimized results^16^. The experimental conditions for ammonia and phenol degradation were optimized using a Box-Behnken design (BBD). Five key parameters - irradiation time, dose of catalyst, concentration of H_2_O_2_, and initial concentrations of ammonia and phenol - were coded with low and high levels in the BBD, as presented in Table 1. Using Eq. (2), the responses were quantified as the ammonia and phenol elimination efficiencies. The low, middle, and high levels’ real values were classified as -1, 0 and 1, respectively. Using Design Expert 7.1.6 software (Stat-Ease Inc., Minneapolis, USA), the 45 runs of the tests were optimized. The statistical association between the independent factors and the dependent response was established using a second-order polynomial model. The linear relationship between the response, the important components, and their interactions is shown in Eq. (2)^16,28^:

2 $$Y={b_o}+\mathop \sum \limits_{{i=1}}^{k} {b_i}{x_i}+\mathop \sum \limits_{{i=1}}^{k} {b_{ii}}x_{i}^{2}+\mathop \sum \limits_{{i=1}}^{k} \mathop \sum \limits_{{j>1}}^{k} {b_{ij}}{x_i}{x_j}+\varepsilon$$

Where Y is the predicted response, b_o_ is the offset term, bi is the coefficient of linear influence, b_ii_ is the coefficient of squared effect, b_ij_ is the coefficient of interaction effect, and ɛ is the random error^16^. Design Expert V.7.0 software was used to perform regression analysis and optimization. The reliability of the model was assessed using ANOVA, with the mean values assigned significant when *p* < 0.05^23^. The three-dimensional response surface analysis of the independent parameters and the dependent factor was used to approximate the optimal data of the operational factors^23^.

## Results and discussion

### Catalysts characterization

#### N-TiO_2_/AC and N-ZnO/AC SEM

**Fig. 3:**
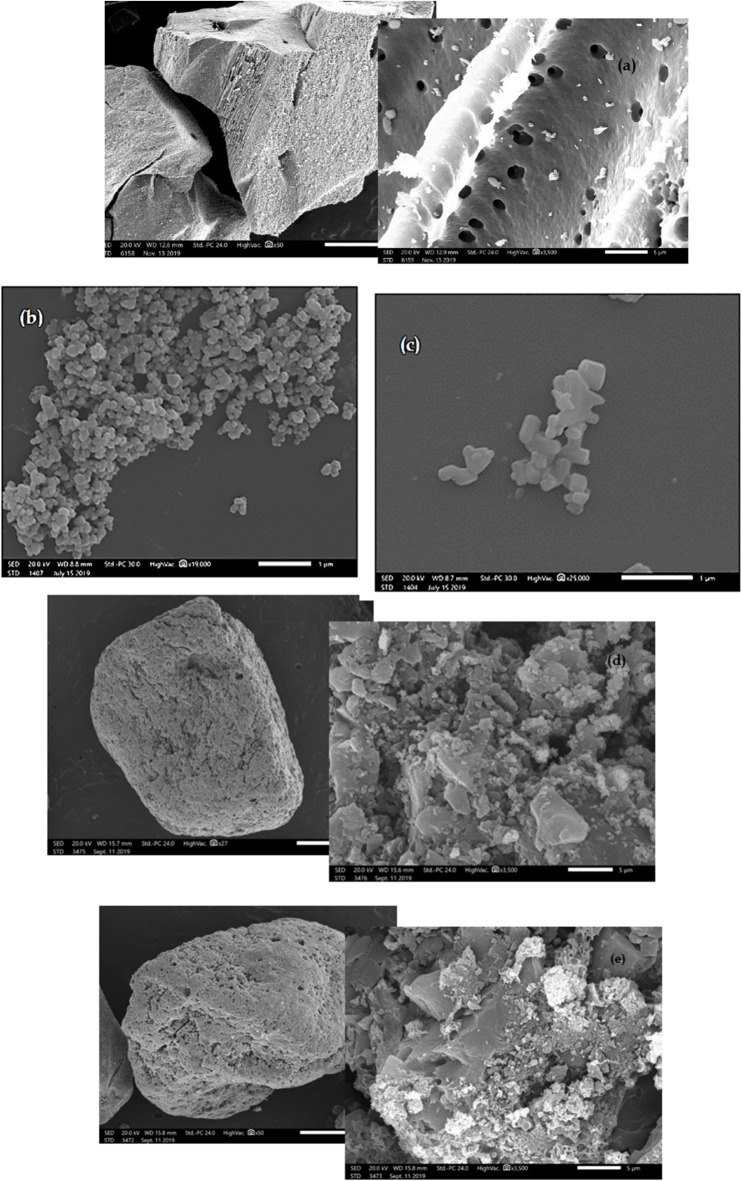
SEM images of (a) GAC, (b) TiO_2_ (c) ZnO, (d) N-TiO_2_/AC and (e) N-ZnO/ AC.

SEM image of granular activated carbon (GAC) (Fig. 3a) reveals its high crystallinity and asymmetric surface morphology, characterized by roughness and the presence of heterogeneous pores. These features provide a favorable environment for the adsorption and deposition of TiO_2_ and ZnO onto the GAC surface and within its pores, which is consistent with previous reports^9,29–32^. The SEM image of TiO_2_ (Fig. 3b) demonstrates well-defined, agglomerated, and spherical TiO_2_ crystals^31,33,34^. The SEM image of N-TiO2/AC (Fig. 3d) shows the consistent participation of TiO2 on the GAC surface and within its bulk after the loading process^32–35^. The N-TiO2/AC catalyst exhibits a mixture of disordered structures relative to GAC particles (Fig. 3a) along with ordered spheres corresponding to homogeneous TiO2 entities (Fig. 3b)^9,30,31^. Moreover, the N-TiO2/AC catalyst displays extremely small-scale spherical patterns that are evenly dispersed over a homogeneous structure, as observed in Fig. 2d^33,34,36^. The SEM image of ZnO (Fig. 3c) confirms its porous structure and network-like appearance^29^. In the case of N-ZnO/AC (Fig. 3e), the GAC particles are uniformly coated with ZnO particles, consistent with previous findings^29,37^. The porous nature of GAC provides favorable sites for the entrapment of ZnO nanoparticles^37^. The zinc oxide particles on the GAC surface exhibit a hexagonal crystalline form^3^. Overall, the SEM analyses confirm that the photocatalysts are uniformly distributed across the GAC matrix, suggesting a synergistic interaction between the active catalyst components and the carbon support^3^.

#### EDX analysis

The presence of carbon (C), nitrogen (N), zinc (Zn), titanium (Ti), and oxygen (O) in both N-ZnO/AC and N-TiO₂/AC composites was confirmed through EDX analysis, as summarized in Table 2. In the EDX spectrum of N-TiO₂/AC, the detection of Ti and Zn elements on the GAC surface indicates successful precipitation of titania and potential residual zinc traces. The presence of carbon suggests that portions of the GAC’s external adsorption sites remained exposed following catalyst loading^3,35^. Additionally, nitrogen incorporation was verified in both N-ZnO/AC and N-TiO₂/AC samples, further supporting the successful doping process, as shown in Table 2.

#### XPS analysis

**Fig. 4:**
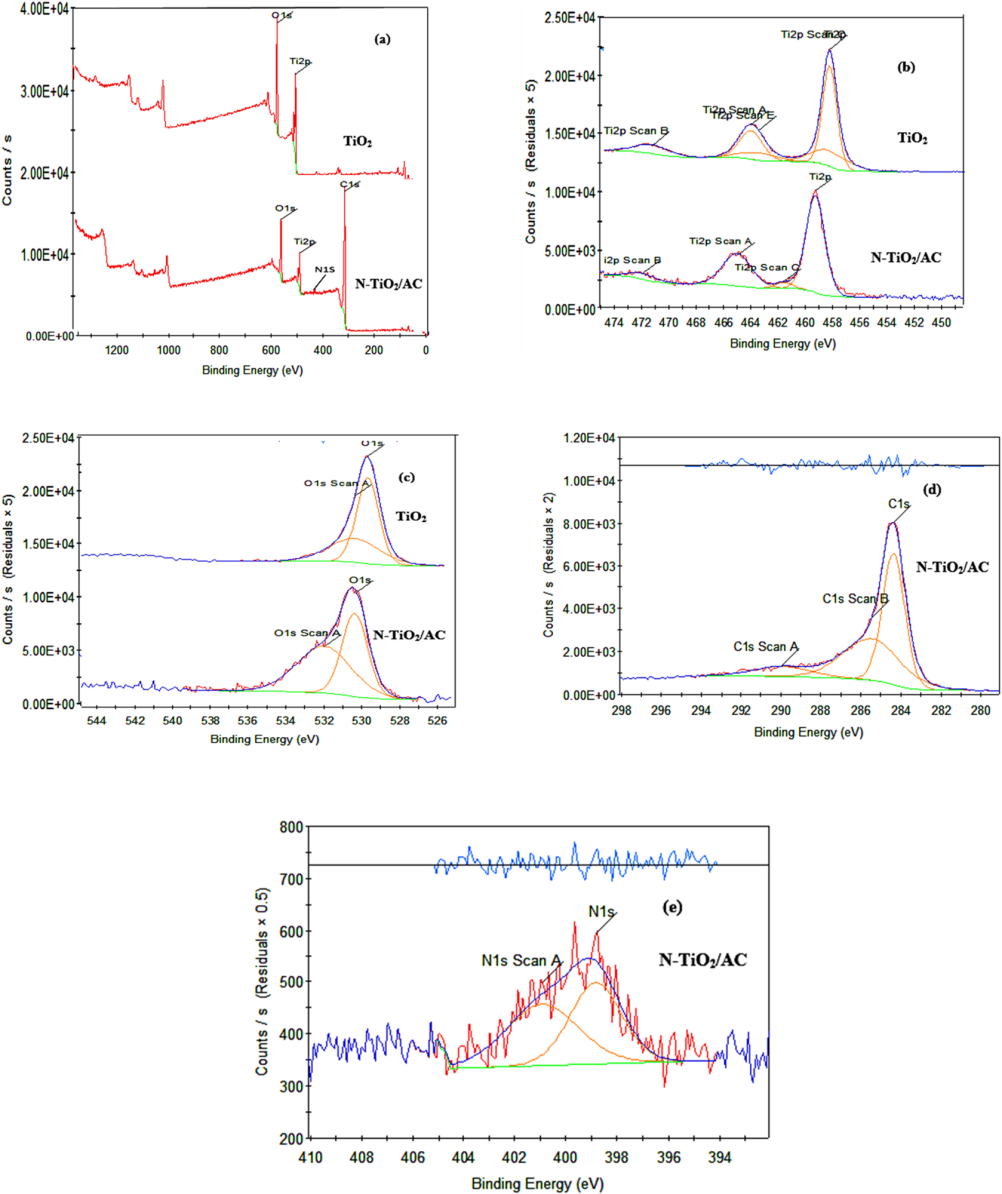
XPS scan of N-TiO_2_/AC and TiO_2_. (a) Survey scan, (b) Ti scan, (c) O scan, (d) C scan and (e) N scan.

The XPS study is effective to detect the possible surface compositions and binding energies of each element in the catalyst and to confirm nitrogen doping in N-TiO_2_/AC and N-ZnO/AC^38^. **For N-TiO**_**2**_**/AC & TiO**_**2**_: Fig. 4 reveals the XPS measurement spectrum for N-TiO_2_/AC and TiO_2_. The four peaks with binding energies of Ti2p, O1s, N1s and C1s at 459.4, 530.1, 398.5 and 285.5ev, respectively, proven the presence of Ti, O, N and C in N-TiO2/AC^39^. While the spectrum of N owned weaker signal, which may be related to the doping nitrogen in a low concentration into the TiO_2_ lattice.

The Ti 2p XPS spectra for **N-TiO**_**2**_**/AC** and TiO2 are exhibited in Fig. 4b. Two peaks appear at 459.4 eV and 466 eV, related to Ti 2p3/2 and Ti 2p1/2, respectively which validates the predominance of titanium in catalysts and is existence as Ti + 4^36,39^. The Ti 2p binding energies may be corresponding to Ti-O, Ti-N, Ti-O-Ti, N-Ti-N and O-Ti-N in N-TiO2/AC^39^. Notably, the peak at **460.5 eV** (Ti 2p₃/₂) is indicative of the formation of **Ti–N bonds**, supporting the successful incorporation of nitrogen into the TiO₂ lattice^17^.

A minor change toward lower binding energies can be seen in the Ti 2p orbital scan of N-TiO_2_/AC. According to previous studies, partial substitution of O^2-^ with N^3-^ causes the production of Ti - N bonds, which affecting the increasing of electron density on Ti ions considering the incorporation of N ions with lower electronegativity^36,40^.

The C is XPS spectra of N-TiO_2_/AC is indicated in Fig. 4d, reveals a dominant peak at 284.3 eV is strongly corresponding to sp2 hybridized carbon binding energy^40^. The C 1 S XPS range has two peaks, 285.5 eV and 289.9 eV, according to curve fitting analysis^35,39^. Since C^4+^ ions are integrated into the bulk structure of TiO_2_, XPS for C 1s peak (289.9 eV) the C 1s could be related to Ti-O-C pattern^39^. C-OH, O=-O, C=O, C-C, C-N, and C-H bonds are responsible for the peaks at 285.5 and 289.9 Ev^17,39,40^.

Using XPS spectra to calculate N is core levels in doped TiO_2_/AC to examine N atoms chemical status (Fig. 4e). Peaks at 402 and 398.5 eV, two peak structures were observed^36^. Bonds at NO and Ti-O-N, O-Ti-N, that are ordinary of N atoms in the TiO_2_ pattern, can be steadily indexed to these peaks^17,20,39^. The latter bonds suggest that nitrogen and carbon have been successfully doped.

**Fig. 5:**
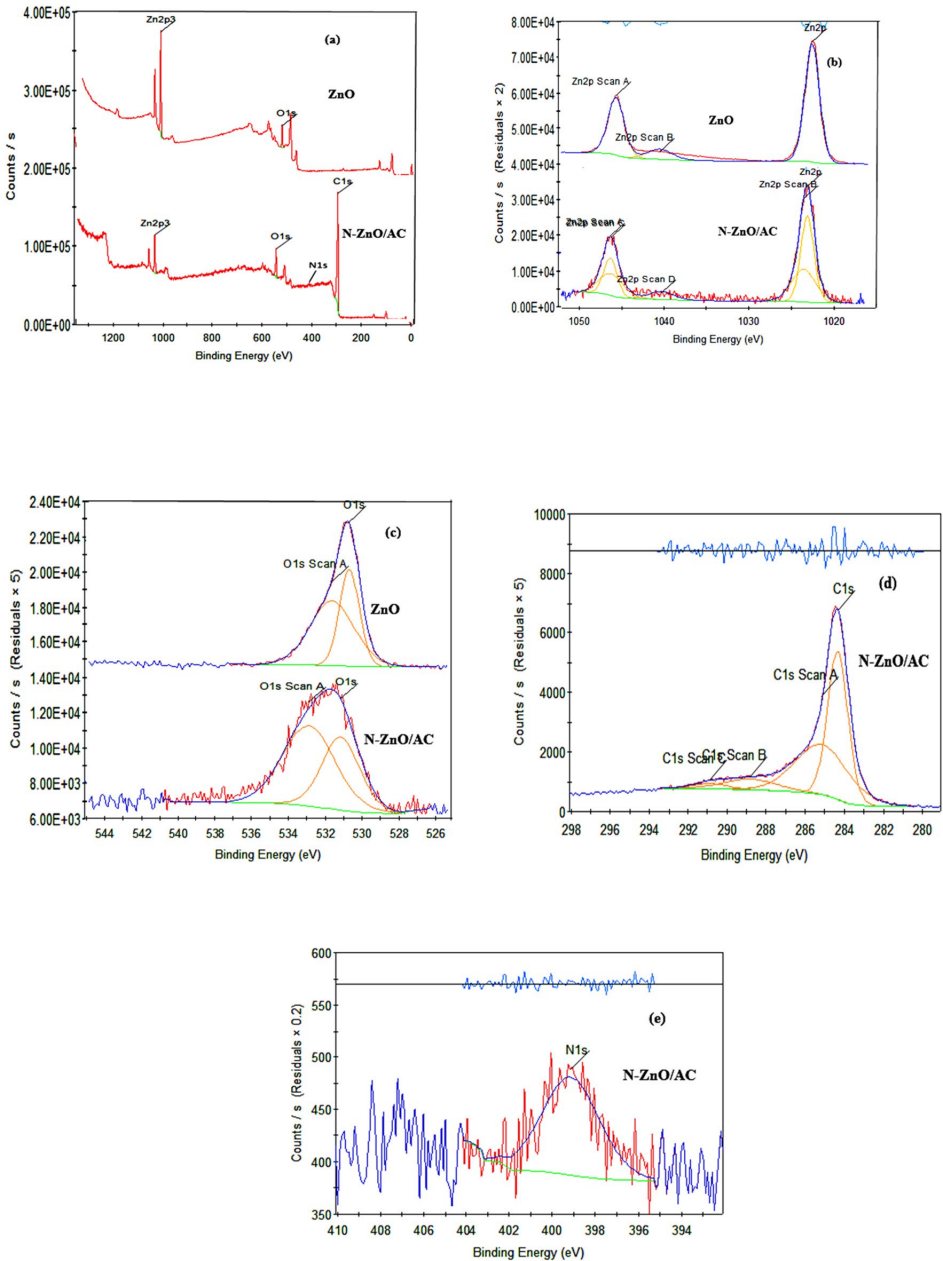
XPS scan of N-ZnO/AC and ZnO. (a) Survey scan, (b) Zn scan, (c) O scan, (d) C scan and (e) N scan.

**For N-ZnO/AC & ZnO**, the XPS spectrum of N-ZnO/AC and ZnO are shown in Fig. 5. Four peaks 1022.3, 533.3, 399.6, and 284.6 ev, which were corresponding to Zn2p, O1s, N1s, and C1s, respectively, indicated the presence of Zn, O, N, and C elements in N-ZnO/AC.

The Zn 2p spectra of N-ZnO/AC and ZnO are presented in Fig. 5b. There are two peaks at 1022.8 eV (Zn-2p3/2) and 1044.6 eV (Zn-2p1/2) related to the Zn^2+^ state for ZnO^41^ and peaks at 1022.3 eV (Zn-2p3/2) and 1044.3 eV (Zn-2p1/2) for N-ZnO/AC. Due to strong interfacial interactions between ZnO and AC could explain this slight change in Zn-2p binding energy peaks^42–44^.

Figure 5c presents the O 1s spectra for the samples, The O-1s binding energy peak (531.4 eV and 533.3 eV) of N-ZnO/AC appears when the binding energy is slightly higher which is indicated (C = O) carbonyl group, epoxy/hydroxyl/metal matrix oxygen (COC)/ C-OH / Zn-O) and atoms of adsorbed water (H-O-H)^42,43^.

Figure 5d shows the C is XPS spectra of N-ZnO/AC, revealing four distinct binding energy peaks. First Peak 284.6 eV is related to C-C linkages in GAC graphitic pattern and non-oxygenated ring C (C-C/C = C), C combined to -OH/ N (C-OH/ C-N)^43,44^. As C-O (epoxy and alkoxy), C = O (carbonyl), and -COOH (carboxylic) oxygen groups have binding energy peaks of 285.8, 288.7, and 291.5 eV, respectively^42–44^.

There is main peak at 399.6 eV detected in the core energy level of N1s with XPS spectroscopy (Fig. 5e) which is related to the N- atoms in N-Zn bond or may be related to -O-Zn-N- bond^15,41^.

#### Raman studies

**Fig. 6:**
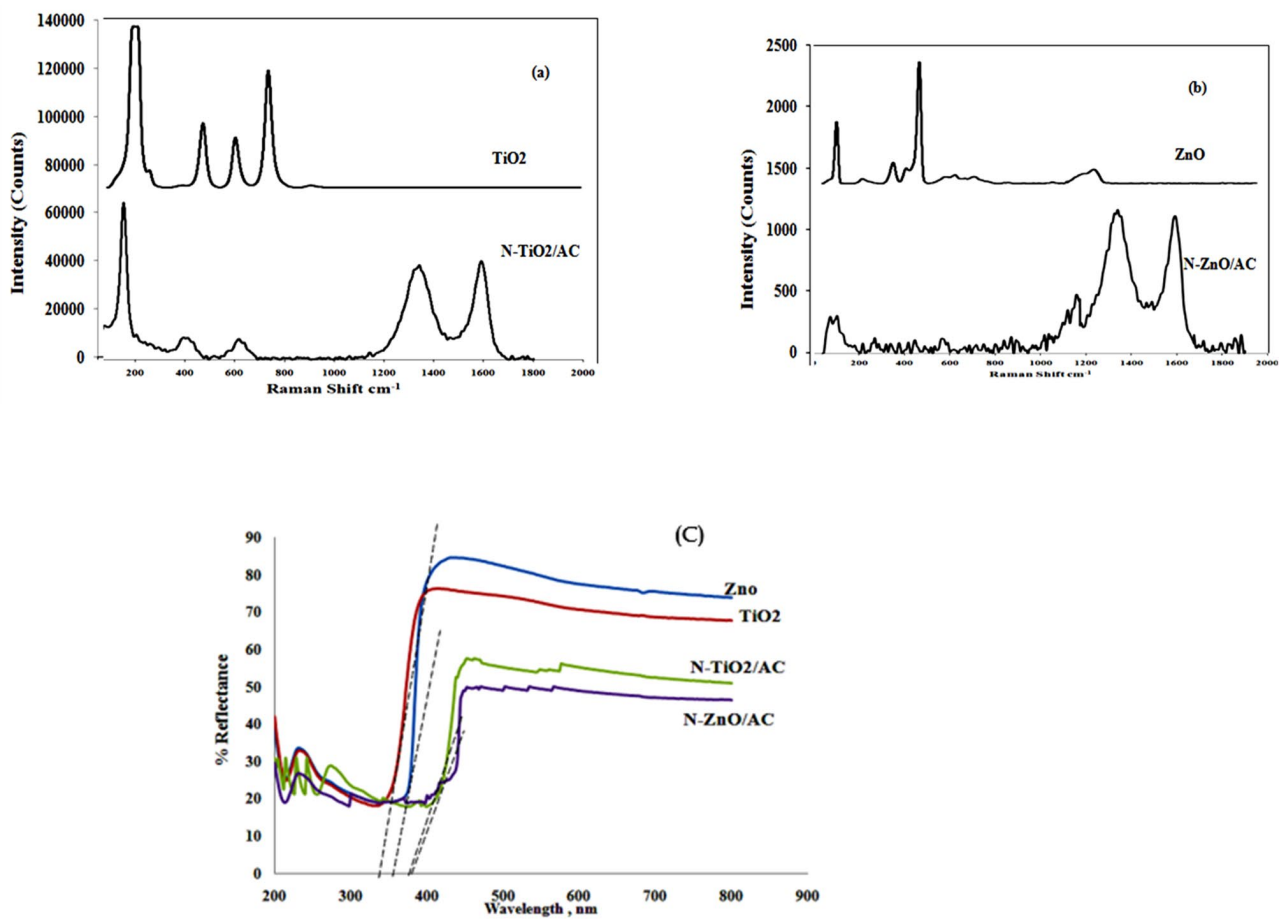
(a) TiO_2_ and N-TiO_2_/AC Raman spectra, (b) ZnO and N-ZnO/AC Raman spectra, C) UV- vis absorption spectra For ZnO, N-ZnO/AC, TiO_2_ and N-TiO_2_/AC photo-catalysts .

Raman analysis was used to determine a substance’s identity and characterise it, particularly for carbonaceous materials^45^. The Raman spectroscopy for N-TiO_2_ /AC, N-ZnO/AC, ZnO and TiO_2_ are shown in Fig. 6a, b.

**TiO**_**2**_ **and N-TiO**_**2**_ **/AC** Raman spectra are shown in Fig. 6a. The TiO_2_ spectrum shows a strong vibration peak at 144.5 cm^− 1^ (A1g), and three flat peaks at 399.6 cm^− 1^ (B1g), 517.3 cm^− 1^ (B1g), and 642.7 cm^− 1^ (A1g), this is assigned to the anatase plane^30^. The two weak peaks at 248.6 cm^− 1^ (A1g) and 321.6 cm^− 1^ (B1g) indicate the existence of a low quantity of brookite^30^. For N-TiO_2_/AC, the low intensity peak at 394 cm^− 1^ is regarding to the vibration of the Ti-N bond, while phonons and photons cause low-frequency and high-frequency scattering at 200 cm^− 1^ and 530 cm^− 1^, respectively.

**For N-ZnO/AC and ZnO**, as presented in Fig. 6b, the Raman peaks corresponding to the ZnO wurtzite- structure as E_2_^low^, E_2_^high^, E_2_^high^ - E_2_^low^ assigned at 120 cm^-1^, 439 cm^-1^ ,339 cm^-1^, respectively. Additionally the Al of transverse phonon (TO) and longitudinal (LO) peaks were assigned at 576 cm^-1^ [Al(LO)] and 383 cm^-1^ [A1(TO)]^38,44–46^. Raman signals below 300 cm^-1^ are thought to be caused by Zn sub-lattice vibrations, whereas higher values are caused by oxygen molecules^46^. Atomic nitrogen existence in ZnO is associated with the other peaks in Fig. 5b N-ZnO/AC at 275 cm^-1^^46^.

**For N-ZnO/AC & N-TiO**_**2**_** /AC:** The typical peaks at 1335 cm^-1^ (labelled as D band) and 1600 cm^-1^ (labelled as G band) indicated the activated carbon in these samples, which has a strong bond to the vibration of sp2-bonded carbon atoms as well as defects and disorder in hexagonal graphitic lattice^30,38,40,44,45^. The magnitude ratio of D-band to G-band (ID/ IG = 1.028) is an indication of the disorder in the crystal pattern related to grain boundaries, vacancies and amorphous carbon^44,46^. These Raman findings are consistent with the XPS analysis, further confirming the structural characteristics of the composite materials.

#### The diffuse reflectance spectroscopy DRS studies

Using UV-vis diffuse reflectance spectra, we investigated the visible light absorption characteristics of N-ZnO/AC, N-TiO2/AC, ZnO, and TiO2. The following equation was utilized for band gap energy estimation^31^:

3 $${E_g}={\mathrm{~}}\frac{{1240}}{{{\lambda _g}}}$$

Where λ_g_ is wavelength by vertical and horizontal portions of the spectra overlapping.

**For N-TiO**_**2**_**/AC and TiO**_**2**_: It is clearly shown in Fig. 6c that TiO_2_ mainly absorbs UV light, while N-TiO_2_/AC can absorb both UV and visible light. As presented in Fig. 6c, pure TiO2 absorption band edge at 380 nm, assigned to a bandgap of 3.26 eV, which is similar to the recorded bandgap of anatase TiO_2_^39,40^.

The band gap energy for N-TiO2/AC was determined to be 3.11 eV, indicating a minor red shift to visible light. The absorption edge is observed at 398 nm. Because carbonaceous materials can absorb visible light and reduce light reflection, the red shift of the N-TiO2/AC absorption band is caused by their existence. As a result, the absorption band’s edge faces the visible light spectrum^36,40^. In general, the production of new energy levels created by doping of non-metal ion can be blamed for the large increase in absorption in the visible light spectrum^39^.

**For N-ZnO /AC and ZnO**, UV-Vis absorption spectra ZnO (Fig. 6c) of as a result to electron moving up from VB to CB, ZnO shows distinct UV field absorption with an absorption edge of about 377 nm and no visible zone absorption (beyond 400 nm). The corresponding bandgap energy, calculated from this absorption edge, is 3.29 eV, which aligns well with previously reported values for ZnO^41,42,44,47^.

For N-ZnO/AC, there is a red shift in the absorption edge from 377 nm to 396 nm and the band-gap energy is measured to be 3.13 eV which is lower than bare ZnO (3.29 eV). As a result, the formation of heterojunctions between ZnO, Nitrogen, and AC extends the absorption to visible wavelengths^42,44,48^. Thus, a shift to lower energy has appeared due to ZnO combination with GAC to which suggests that the inclusion of carbon causes an increase in the electric surface charge of the oxide inside the matrix, which could lead to changes in the fundamental process of electron-hole pair formation during irradiation^47,49^. Also, doping of nitrogen lead to Lattice defects formed depending on this shift. This certifies the enhancement of photo response for the synthesized catalyst in Vis zone as well as UV zone of the spectrum^41^.

### Evaluation of degradation efficiency for N-ZnO/AC and N-TiO2/AC catalysts

#### Photo-catalytic activities for N-ZnO/AC and N-TiO_2_/AC catalysts

**Fig. 7:**
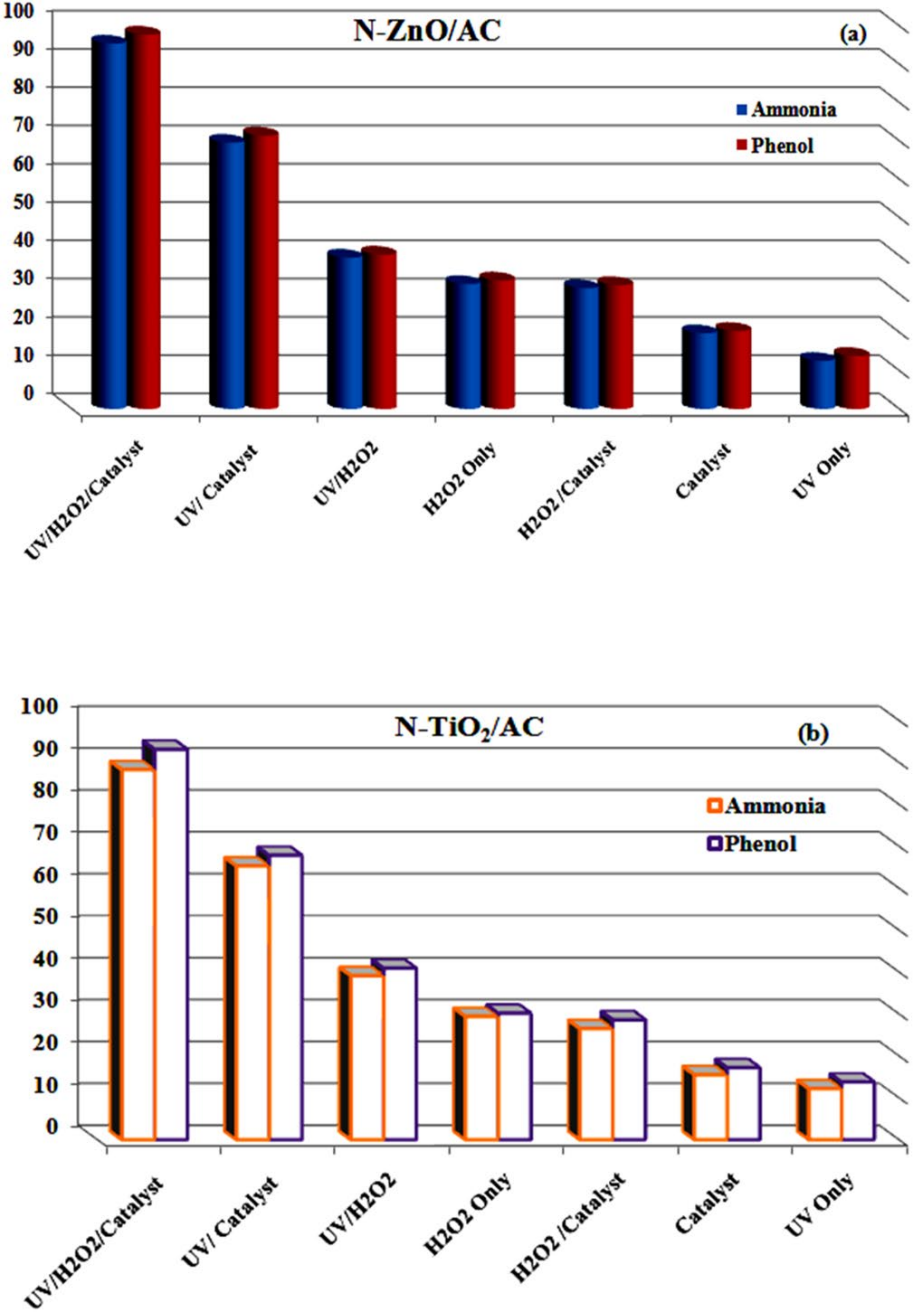
Effect of various oxidation processes for ammonia and phenol degradation: (a) N-ZnO/AC, (b) N-TiO_2_/AC: [C_o(Ammonia)_ 300 mg/L, C_o(Phenol)_ 100 mg/L, pH 6.2, dose 0.5 g/L, flow rate 8 L/min, H_2_O_2_ 10 mM ,45 W, 180 min].

To estimate the degradation efficiency, the effect of each factor involved in photo-degradation process (UV/catalyst/H_2_O_2_) was studied. The degradation efficiency of each parameter including photolysis processes by UV, photo-catalyst (N-ZnO/AC and N-TiO_2_/AC), H_2_O_2_, UV/ Catalyst, UV/ H_2_O_2_ and hybrid advanced oxidation process (UV/Catalyst/ H_2_O_2_) in the degradation efficiency of ammonia and phenol at the same conditions was shown in Fig. 7a, b.

In the absence of UV and H2O2 the ammonia and phenol photo-degradation was minimal with degradation not exceeding 20% for either N-ZnO/AC or N-TiO₂/AC catalysts. This limited removal may be attributed primarily to adsorption onto the catalyst surface. When the UV Upon the introduction of UV light, the removal efficiency of both pollutants increased. A further enhancement was observed when UV irradiation and H₂O₂ were combined in the presence of the catalyst, resulting in a significant improvement in degradation efficiency.

The addition of H2O2 to a photo-catalytic device is one of the most popular techniques to raise the concentration of ·OH. In fact, the process can produce more ·OH by adding H_2_O_2_ without raising light intensity, which boosts UV photonic efficiency and enhances process efficiency and cost-effectiveness^10,50^. Increasing the generation of ·OH, decreasing electron-hole pain recombination, and improving photonic efficiency are other effects of the addition of H_2_O_2_^50^. Moreover, H_2_O_2_ can interact with the valence band, leading to the formation of hydroperoxyl radicals (HO_2_·), which accelerate the degradation of pollutants^50^.

4 $${{\mathrm{H}}_2}{{\mathrm{O}}_2}+{\mathrm{~}}{{\mathrm{h}}^+} \to {{\mathrm{H}}^+}+{\mathrm{HO}}_{2}^{ \cdot }{\mathrm{~}}$$

As shown from Fig. 7a, b for the two catalysts N-ZnO/AC and N-TiO_2_/AC, ammonia and phenol degradation efficiency is significantly enhanced by the integration of H_2_O_2_/UV with Catalyst/UV which was (97.606% and 93.082% phenol) and (95.261% and 88.237% ammonia) for both N-ZnO/AC and N-TiO_2_/AC, respectively. Based on these results, all the coming trials were carried out using UV/Catalyst/ H_2_O_2_ system.

#### Effect of cyclic flow rate

**Fig. 8:**
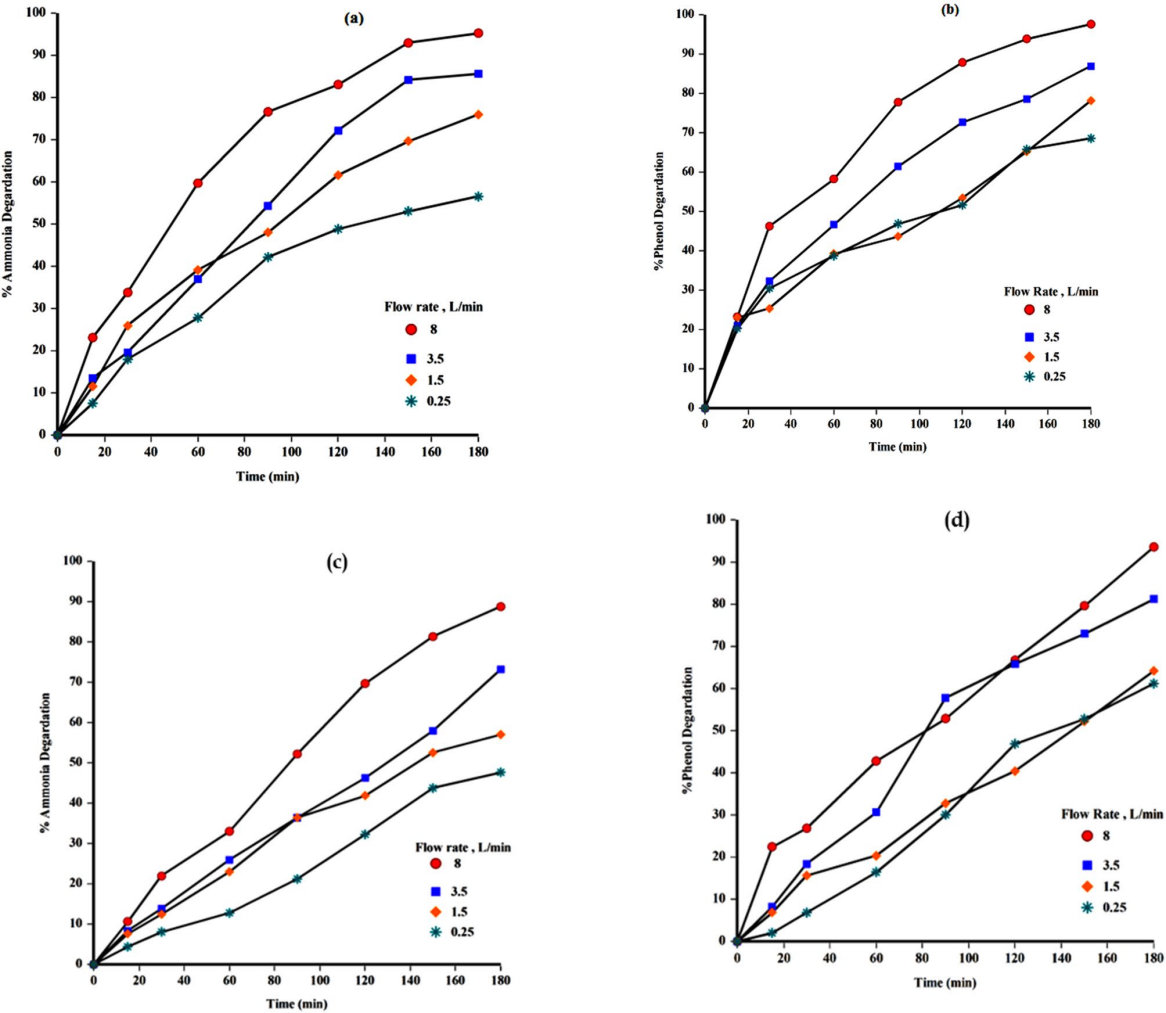
(a) Effect of flow rate for N-ZnO/AC catalyst- ammonia, (b) effect of flow rate for N-ZnO/AC catalyst- phenol, (c) effect of flow rate for N- TiO_2_/AC catalyst- ammonia, (d) effect of flow rate for TiO_2_/AC catalyst- phenol : [C_o(Ammonia)_ 300 mg/L, C_o(Phenol)_ 100 mg/L, pH 6.2, dose 0.5 g/L, H_2_O_2_ 10 mM ,45 W, 180 min].

The impact of varying flow rates on the photocatalytic degradation performance is illustrated in Fig. 8a–d for the N-ZnO/AC and N-TiO₂/AC catalysts, respectively. The results indicated that increasing flow rate from 0.25 to 8 L/min increased the efficiency degradation. At the highest flow rate of 8 L/min, ammonia removal reached 95.26% and 88.75% for N-ZnO/AC and N-TiO₂/AC, respectively, while phenol degradation achieved 97.61% and 93.63% for the same catalysts. These improvements can be attributed to the influence of flow rate on photocatalytic performance, primarily through its effect on convective mass transfer and residence time within the reactor.

At high flow rates, convective mass transfer plays a critical role in facilitating the degradation reaction, while at lower flow rates, the reaction becomes constrained by residence time^51^. The observed increase in degradation efficiency with rising flow velocity suggests that convective transport is the dominant limiting factor governing the mass transfer-driven degradation process^51^. Based on these observations, the optimal flow rate ammonia and phenol degradation using N-ZnO/AC and N-TiO2/AC catalysts was determined to be 8 L/min, which was subsequently employed for all subsequent trails.

#### Effect of pH

**Fig. 9:**
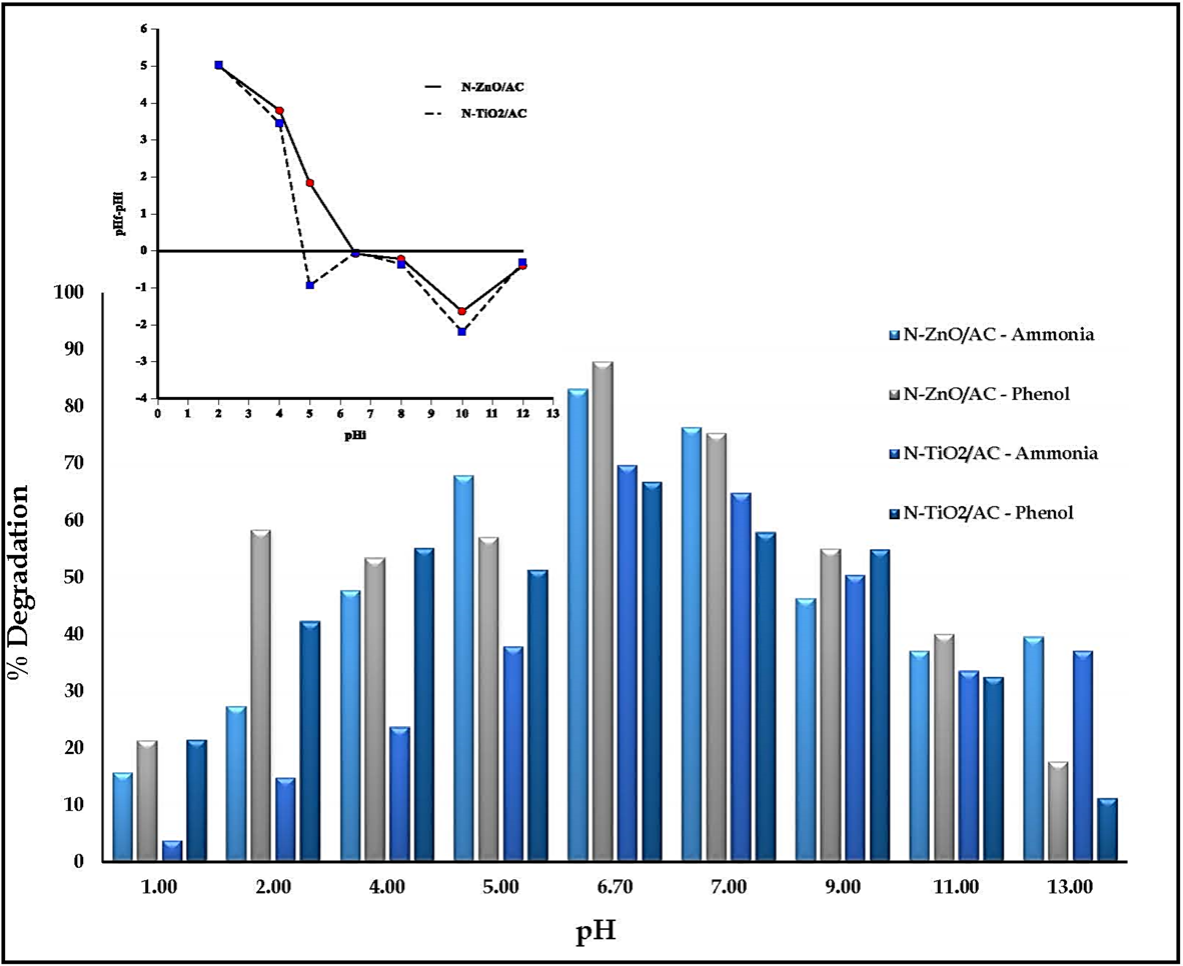
Effect of pH on ammonia and phenol degradation: [C_o(Ammonia)_ 300 mg/L, C_o(Phenol)_ 100 mg/L, dose 0.5 g/L, flow rate 8 L/min, H_2_O_2_ 10 mM ,45 W, 120 min].

As shown in Fig. 9, the binary solution at neutral pH (6.7) revealed the highest degradation efficiencies for both N-ZnO/AC and N-TiO₂/AC catalysts. For N-ZnO/AC catalyst, the degradation efficiencies were approximately 83% and 88%, respectively. Similarly, for N-TiO2/AC catalyst, the degradation efficiencies of ammonia and phenol reached 70% and 67%, respectively. Moreover, the pH at the point of zero charge (pH_ZPC_) was determined for N-ZnO/AC and N-TiO_2_/AC catalysts by comparing the pH values before and after immersing them in potassium nitrate. The pH_ZPC_ values were found to be 6.4 for N-ZnO/AC and 5.4 for N-TiO_2_/AC, consistent with values reported in the literature for ZnO/AC and TiO_2_/AC catalysts^49,52^. Ammonia nitrogen degradation in the binary solution was decreased under acidic conditions due to strong repulsion between H^+^ and NH_3_^+^ molecules^53,54^. The presence of OH^-^ negative charges at higher pH values enhances ammonia decomposition, which decreases with further increases in solution pH. On the other hand, degradation of phenols in acidic conditions (pH < 3) is undesirable but increasing solution pH enhances solution photolysis. At higher pH levels, phenol ions transform into phenolate anions, leading to coulombic repulsion between the negatively charged catalyst surface and hydroxide anions^55^. Consequently, neutral pH was identified as the optimal condition for the simultaneous degradation of ammonia and phenol, as shown in Fig. 9.

Based on our previous results, it is obvious that N-ZnO/ AC catalyst exhibits better degradation efficiency for both phenol and ammonia compared to N-TiO_2_/AC catalyst. To further optimize the efficiency of the N-ZnO/AC catalyst and evaluate the key and mutual effects of the operating parameters on the photo-degradation mechanism, design of experiment tools were utilized.

### Model finding of ammonia and phenol degradation using N-ZnO/AC catalyst

#### N-ZnO/AC degradation model and ANOVA analysis

To establish meaningful relationship between the degrading efficiency (dependent variable) and independent factors in the photo-catalytic system, a response surface methodology based on a Box-Behnken design was investigated. Design-Expert software was used to generate the models for ammonia and phenol degradation, as expressed in Eqs. (5) and (6), respectively (Table 3).

The design equations as mentioned below:

5 $$\begin{aligned} \% {\text{ Y }}\left( {{\mathrm{Ammonia}}} \right) & \,=\,{\mathrm{89}}.{\mathrm{97}}\,+\,{\mathrm{9}}.{\mathrm{43}}*{\text{ }}{{\mathrm{X}}_{\mathrm{1}}}\,+\,{\mathrm{8}}.{\mathrm{14}}*{\text{ }}{{\mathrm{X}}_{\mathrm{2}}}\,+\,{\mathrm{4}}.{\mathrm{15}}*{\text{ }}{{\mathrm{X}}_{\mathrm{3}}} - {\mathrm{5}}.{\mathrm{24}}*{\text{ }}{{\mathrm{X}}_{\mathrm{4}}}\,+\,0.{\mathrm{59}}*{\text{ }}{{\mathrm{X}}_{\mathrm{5}}} \\ & \;\;\; - {\mathrm{2}}.0{\mathrm{6}}*{\text{ }}{{\mathrm{X}}_{\mathrm{1}}}*{\text{ }}{{\mathrm{X}}_{\mathrm{2}}}\,+\,{\mathrm{1}}.{\mathrm{23}}*{\text{ }}{{\mathrm{X}}_{\mathrm{1}}}*{\text{ }}{{\mathrm{X}}_{\mathrm{3}}}\,+\,{\mathrm{2}}.{\mathrm{81}}*{\text{ }}{{\mathrm{X}}_{\mathrm{1}}}*{\text{ }}{{\mathrm{X}}_{\mathrm{4}}}\,+\,0.{\mathrm{87}}*{\text{ }}{{\mathrm{X}}_{\mathrm{1}}}*{\text{ }}{{\mathrm{X}}_{\mathrm{5}}} \\ & \;\; - {\mathrm{5}}.{\mathrm{22}}*{\text{ }}{{\mathrm{X}}_{\mathrm{2}}}*{\text{ }}{{\mathrm{X}}_{\mathrm{3}}}\,+\,{\mathrm{2}}.{\mathrm{82}}*{\text{ }}{{\mathrm{X}}_{\mathrm{2}}}*{\text{ }}{{\mathrm{X}}_{\mathrm{4}}}\,+\,0.0{\mathrm{84}}*{\text{ }}{{\mathrm{X}}_{\mathrm{2}}}*{\text{ }}{{\mathrm{X}}_{\mathrm{5}}} - {\mathrm{2}}.{\text{82 }}*{\text{ }}{{\mathrm{X}}_{\mathrm{3}}}*{\text{ }}{{\mathrm{X}}_{\mathrm{4}}} \\ & \;\; - {\mathrm{1}}.{\text{65 }}*{\text{ }}{{\mathrm{X}}_{\mathrm{3}}}*{\text{ }}{{\mathrm{X}}_{\mathrm{5}}} - {\mathrm{1}}.0{\mathrm{5}}*{\text{ }}{{\mathrm{X}}_{\mathrm{4}}}*{\text{ }}{{\mathrm{X}}_{\mathrm{5}}} - {\mathrm{4}}.{\mathrm{11}}*{\text{ }}{{\mathrm{X}}_{\mathrm{1}}}^{{\mathrm{2}}} - {\mathrm{2}}.{\mathrm{54}}*{\text{ }}{{\mathrm{X}}_{\mathrm{2}}}^{{\mathrm{2}}} - {\text{ 5}}.{\mathrm{71}}*{\text{ }}{{\mathrm{X}}_{\mathrm{3}}}^{{\mathrm{2}}} \\ & \;\; - {\mathrm{1}}.{\mathrm{84}}*{\text{ }}{{\mathrm{X}}_{\mathrm{4}}}^{{\mathrm{2}}}\,+\,0.{\text{72 }}*{\text{ }}{{\mathrm{X}}_{\mathrm{5}}}^{{\mathrm{2}}} \\ \end{aligned}$$

6 $$\begin{aligned} \% {\text{ Y }}\left( {{\mathrm{Phenol}}} \right)\, & =\,{\mathrm{9}}0.{\mathrm{68}}\,+\,{\mathrm{7}}.{\text{81 }}*{{\mathrm{X}}_{\mathrm{1}}}\,+\,{\mathrm{7}}.{\mathrm{87}}*{{\mathrm{X}}_{\mathrm{2}}}\,+\,{\mathrm{5}}.{\mathrm{49}}*{{\mathrm{X}}_{\mathrm{3}}} - \,0.{\mathrm{21}}*{{\mathrm{X}}_{\mathrm{4}}} - {\mathrm{5}}.0{\mathrm{3}}*{{\mathrm{X}}_{\mathrm{5}}} - {\mathrm{2}}.0{\mathrm{8}}*{{\mathrm{X}}_{\mathrm{1}}}*{{\mathrm{X}}_{\mathrm{2}}}\, \\ & \;\;+\,0.{\text{84 }}*{{\mathrm{X}}_{\mathrm{1}}}*{{\mathrm{X}}_{\mathrm{3}}} - \,0.{\mathrm{64}}*{{\mathrm{X}}_{\mathrm{1}}}*{{\mathrm{X}}_{\mathrm{4}}}\,+\,{\mathrm{5}}.0{\text{2 }}*{{\mathrm{X}}_{\mathrm{1}}}*{{\mathrm{X}}_{\mathrm{5}}} - {\mathrm{2}}.{\text{92 }}*{{\mathrm{X}}_{\mathrm{2}}}*{{\mathrm{X}}_{\mathrm{3}}}\,+\,0.0{\mathrm{72}}*{{\mathrm{X}}_{\mathrm{2}}}*{{\mathrm{X}}_{\mathrm{4}}}\, \\ & \;\;+\,{\mathrm{3}}.{\text{63 }}*{{\mathrm{X}}_{\mathrm{2}}}*{{\mathrm{X}}_{\mathrm{5}}} - 0.{\text{22 }}*{{\mathrm{X}}_{\mathrm{3}}}*{{\mathrm{X}}_{\mathrm{4}}} - {\mathrm{1}}.{\mathrm{1}}0{\mathrm{1}}*{{\mathrm{X}}_{\mathrm{4}}}*{{\mathrm{X}}_{\mathrm{5}}} - {\mathrm{1}}.{\mathrm{27}}*{\text{ }}{{\mathrm{X}}_{\mathrm{4}}}*{\text{ }}{{\mathrm{X}}_{\mathrm{5}}} - {\mathrm{3}}.0{\mathrm{5}}*{\text{ }}{{\mathrm{X}}_{\mathrm{1}}}^{{\mathrm{2}}} \\ & \;\; - \,{\mathrm{3}}.{\text{23 }}*{\text{ }}{{\mathrm{X}}_{\mathrm{2}}}^{{\mathrm{2}}} - {\mathrm{2}}.{\mathrm{28}}*{\text{ }}{{\mathrm{X}}_{\mathrm{3}}}^{{\mathrm{2}}}\,+\,0.{\mathrm{65}}*{\text{ }}{{\mathrm{X}}_{\mathrm{4}}}^{{\mathrm{2}}}\,+\,{\mathrm{1}}.{\mathrm{12}}*{{\mathrm{X}}_{\mathrm{5}}}^{{\mathrm{2}}} \\ \end{aligned}$$

Model validation was performed using analysis of variance (ANOVA) at a 95% confidence level, as shown in Table 4. The F-value for the ammonia degradation model was 92.79, indicating its significance. Similarly, the F-value for the phenol degradation model was 120.2, indicating its significance. With a probability of only 0.01%, such high “Model F-Values” are unlikely to occur due to noise. The “Lack of Fit (LOF) F-value” for the ammonia degradation model was 2.76, indicating a 16.74% chance of such a large “Lack of Fit F-value” occurring due to noise. The non-significant LOF P-value (0.1674 > 0.05 and Fcal = 2.76 Fc = 2.78) confirms the statistical soundness of the chosen model^56–58^. Similarly, the “Lack of Fit F-value” for the phenol degradation model was 0.37, indicating a 94.11% chance of such a large “Lack of Fit F-value” occurring due to noise. The non-significant LOF P-value (0.9411 > 0.05 and Fcal = 0.37 Fc = 2.78), confirms the statistical robustness of the selected model. This non-significant LOF suggests that the fitted model matches the data well^56–58^.

Correlation coefficients (R^2^) were employed to assess the goodness of fit of the models to the experimental results. R^2^ ranges from 0 to 1, with higher values indicating better agreement between the regression equation and the sample data^58^. The R^2^ values for the ammonia degradation model (R^2^ = 0.9872) and the phenol degradation model (R^2^ = 0.9901) indicate good agreement between the experimental and predicted data. However, adjusted R² is often considered a more reliable indicator of model adequacy, as it accounts for the number of variables included and prevents artificial inflation of R² due to the addition of statistically insignificant terms^59–61^. The adjusted-R^2^ values for the ammonia degradation model (adjusted-R^2^ = 0.9766) and the phenol degradation model (adjusted-R^2^ = 0.9819) show strong agreement with their respective R² values, confirming the reliability of the models. As presented in Table 4, the variables Time (X₁), Catalyst Dose (X₂), and H₂O₂ Concentration (X₃) had the most pronounced positive effects on the degradation efficiencies of both ammonia and phenol. But, for the phenol degradation model, the phenol initial concentration (X5) had a significant negative impact on phenol degradation efficiency, while initial ammonia concentration (X4) had no significant impact on phenol degradation efficiency.

**Fig. 10:**
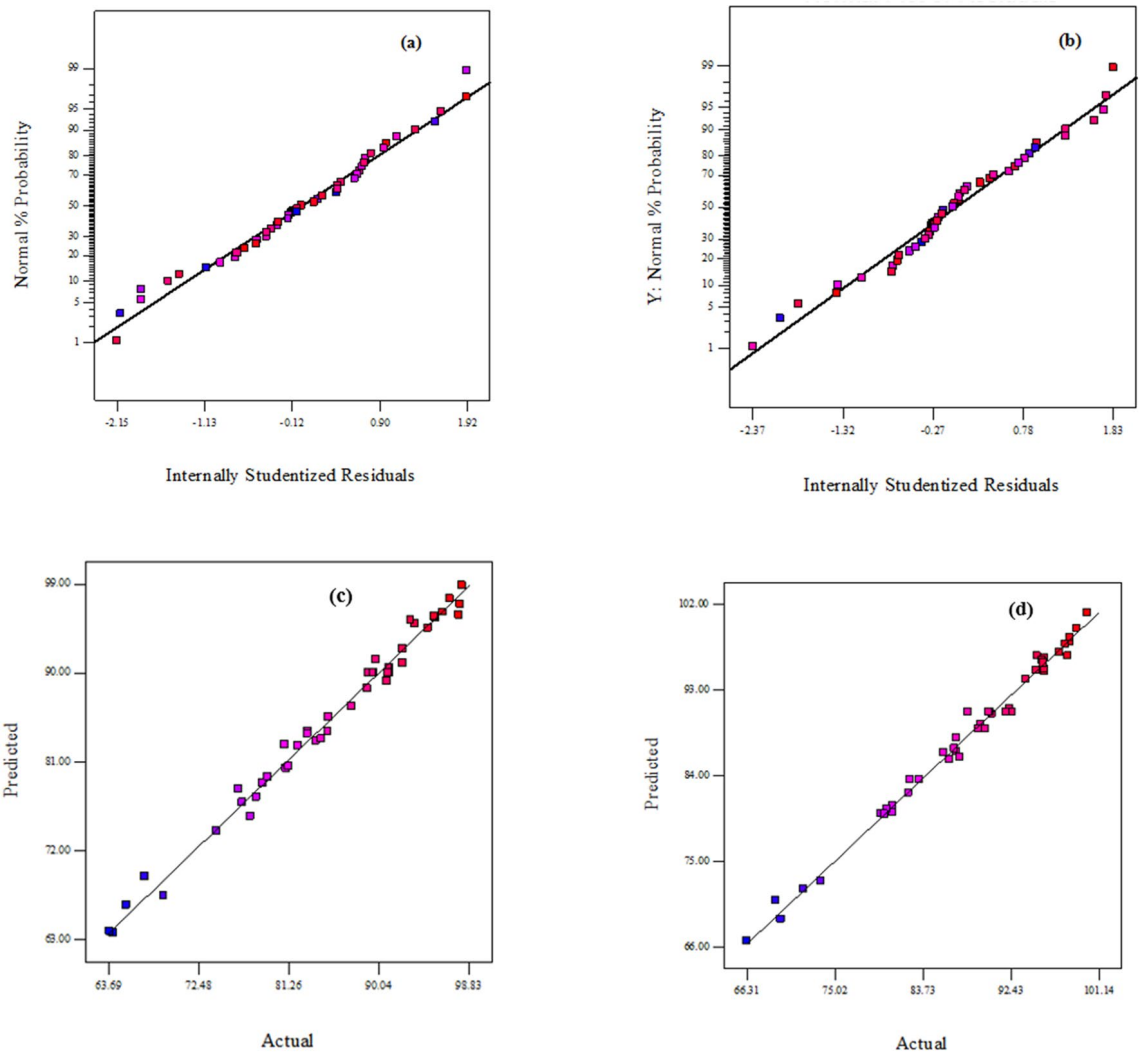
(a) Ammonia normal probability plot, (b) phenol normal probability plot, (c) ammonia plot of the predicted versus the actual degradation efficiency, (d) phenol plot of the predicted versus the actual degradation efficiency.

Normal probability curves (Fig. 10a, b) for both models indicate that the collected data follows a normal distribution along a straight line. The relatively low coefficients of variation (CV) for the ammonia degradation model (1.7%) and the phenol degradation model (1.33%) validate the accuracy of the measured data and the models. The actual values for both models closely match the expected values (Fig. 10c, d), indicating their adequacy and significance^59^.

#### RSM results

Based on RSM, three-dimensional response surface plots utilized to graphically represent the influence of independent variables and their interactions on the degradation of ammonia and phenol to verify the optimum value for ammonia and phenol removal.

**Fig. 11:**
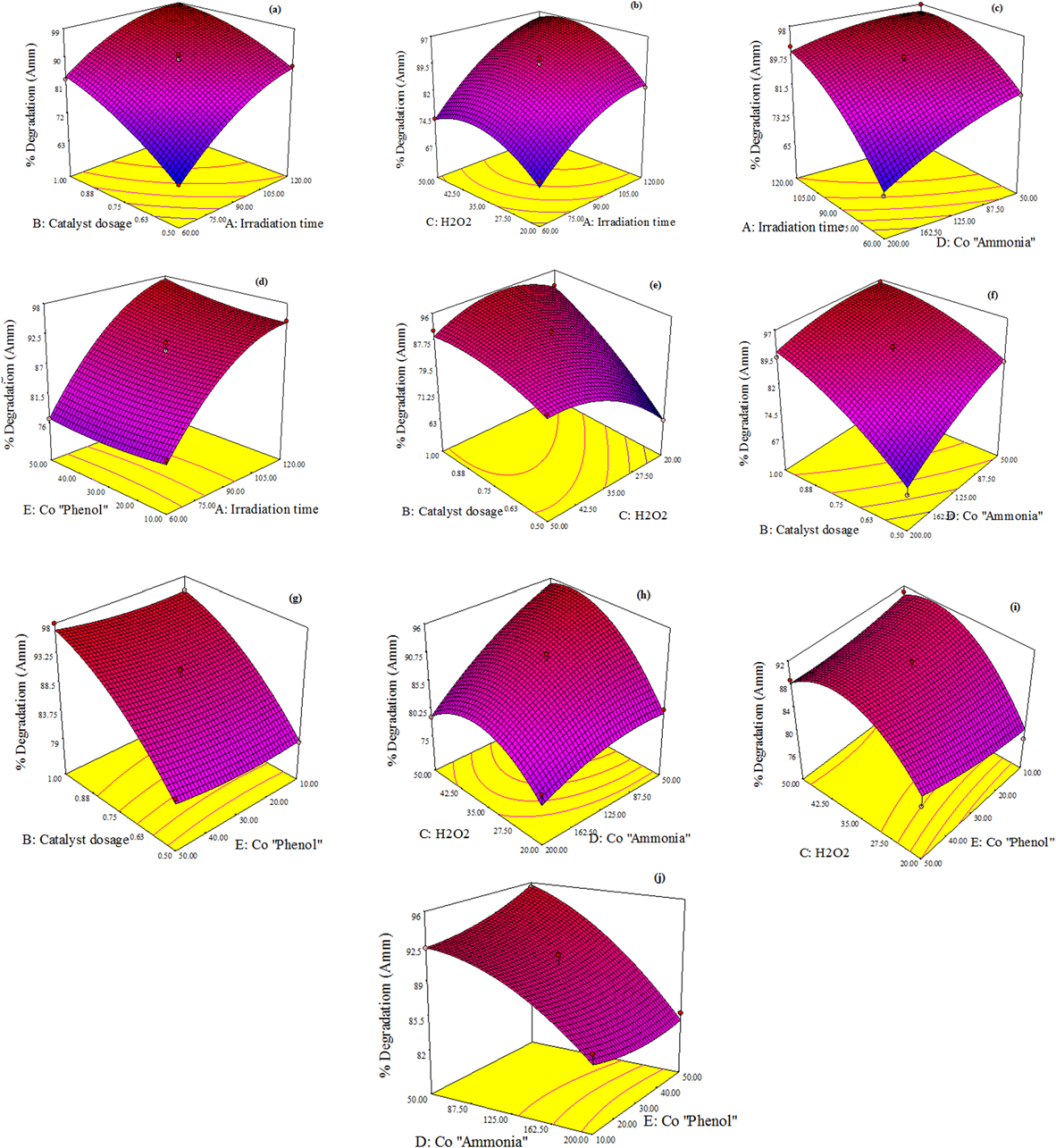
3D response surface plots for effects of process variables on ammonia photo-degradation percent by N-ZnO/AC catalyst.

**Fig. 12:**
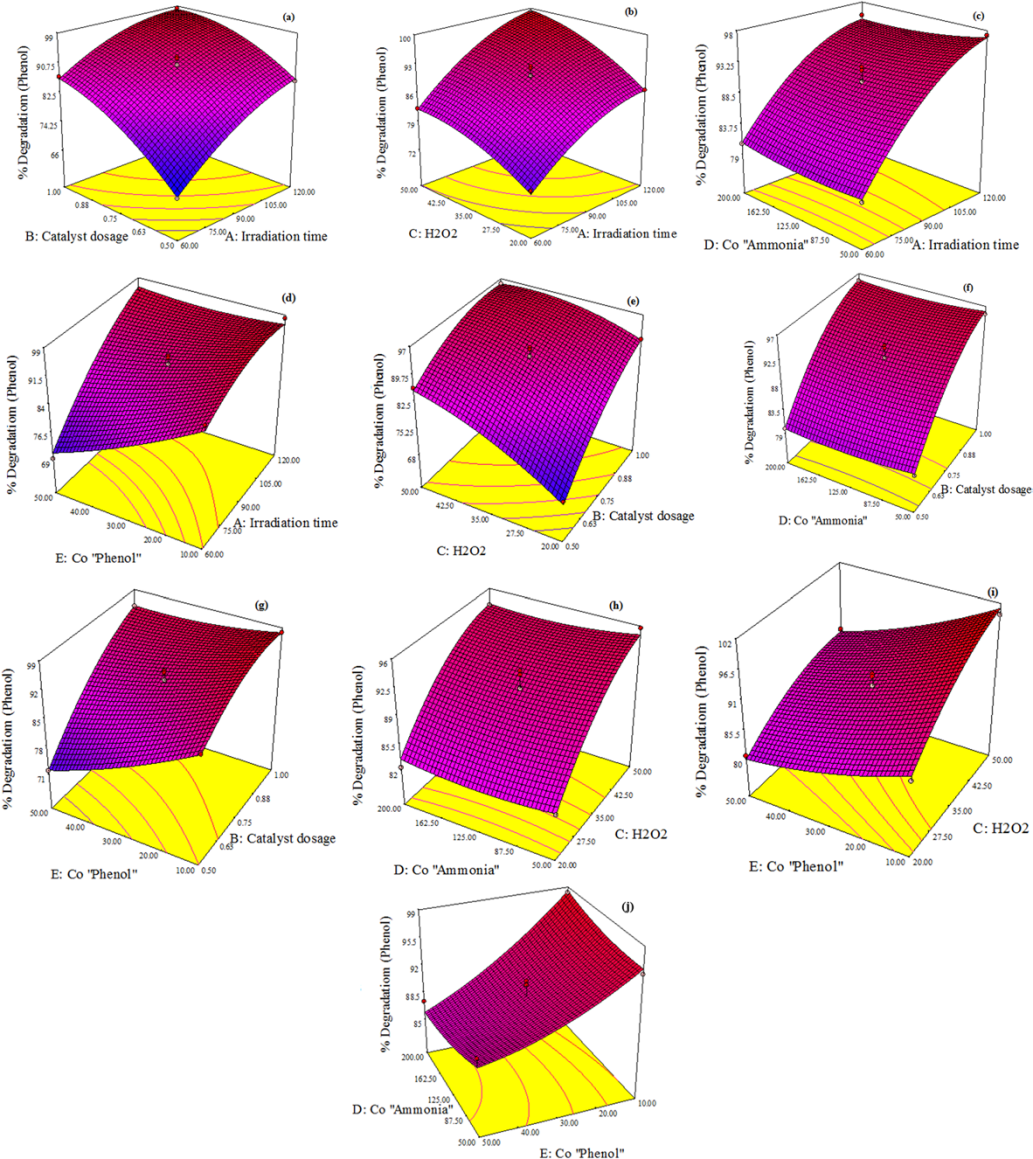
3D response surface plots for effects of process variables on phenol photo-degradation percent by N-ZnO/AC catalyst.

Figures 11a and 12a reveal the interaction between irradiation time and catalyst dose. The common interaction curve for two variables proves that by consequently increasing the catalyst and reaction time an improvement of the degradation performance was occurred. As irradiation time increases, the number of photons absorbed on the surface of the catalyst increases, thereby enhancing the photo-catalytic process^62^. Similarly, increasing the catalyst dosage boosts the photocatalytic efficiency, as a higher quantity of catalyst generates more charge carriers, which in turn accelerates the degradation of the target compound^16^.

The interaction between irradiation time and H₂O₂ concentration is illustrated in Figs. 11b and 12b, corresponding to ammonia and phenol degradation efficiencies, respectively. For ammonia, the degradation efficiency increases with H₂O₂ concentration up to 35 mM. Beyond this threshold, a decline in performance is observed, attributed to the conversion of reactive hydroxyl radicals (·OH) into less reactive hydroxide ions (OH⁻)^7^. But for the phenol degradation model shows a consistent improvement in removal efficiency with increasing H₂O₂ dosage and irradiation time. The elevated H₂O₂ concentration promotes greater ·OH generation, thereby enhancing the photocatalytic degradation of phenol^28^.

Figures 11c, d and 12c, d characterized the interaction between irradiation time and the initial concentrations of ammonia and phenol, respectively, in relation to their degradation efficiencies. For ammonia, an increase in its initial concentration leads to a reduction in degradation efficiency. Similarly, higher initial concentrations of phenol result in decreased phenol degradation efficiency. This decline at elevated concentrations may be attributed to the limited availability of active sites on the catalyst surface, which become saturated under high substrate loads^63,64^. Under constant catalyst dosage and fixed H_2_O_2_ concentration, the generation of reactive species such as hydroxyl radicals (·OH) and superoxide anions (O_2_^–^) remains unchanged^63^. Consequently, the degradation efficiency diminishes as the initial concentrations of ammonia and phenol increase. For degradation of ammonia, increasing initial concentration of phenol had a slight effect on degradation of ammonia for example when irradiation time is 60 min and initial phenol concentration 50 ppm ammonia removal is 76% and at time 60 min and initial phenol concentration 10 ppm ammonia removal is 78%. For degradation of phenol, increasing initial concentration of ammonia doesn’t have any effect on degradation of phenol for example when irradiation time is 60 min and initial ammonia concentration 200 ppm phenol removal is 80% and at time 60 min and initial ammonia concentration 50 ppm phenol removal is 79%.

Catalyst Dose and H_2_O_2_ concentration interaction was shown in Figs. 11e and 12e for both ammonia and phenol degradation efficiency respectively. By increasing dose and increasing H_2_O_2_ concentration till 35 mM the removal of ammonia increased but by increasing H_2_O_2_ furthermore 35 mM ammonia removal efficiency decreased as at Dose 0.5 g and H_2_O_2_ 50 mM ammonia removal is 80% but at Dose 0.75 g and H_2_O_2_ 35 mM ammonia removal is 89%. For phenol degradation is increased by increasing two variables catalyst dose and H_2_O_2_.

Figures 11f, g and 12f, g illustrate the impact of catalyst does and initial concentration of ammonia and phenol on the degradation efficiency for the both pollutants. We could reach to optimum ammonia removal by increasing catalyst dose and decreasing initial ammonia concentration as the optimum value for ammonia removal was 96% at dose 1 g/L and initial ammonia concentration 50 ppm but for phenol degradation as we mentioned above initial ammonia concentration parameter has no effect on phenol removal. The optimum phenol removal was reached by increasing catalyst dose and decreasing initial phenol concentration as the optimum value for ammonia removal was 97% at dose 1 g/L and initial phenol concentration 10 ppm but for ammonia degradation as we mentioned before initial phenol concentration parameter has a slight effect on ammonia degradation as at dose 1 g/L and C_o(Phenol)_ = 100 ppm ammonia removal is 95% and at dose 1 g/L, and C_o(Phenol)_ = 50 ppm ammonia removal is 98% .

Figures 11h, i and 12h, i illustrate the interaction between H_2_O_2_ concentration and the initial concentrations of ammonia and phenol, respectively, in relation to their degradation efficiencies. In the ammonia degradation model, H_2_O_2_ exhibits limited influence at higher initial ammonia concentrations. However, at lower ammonia levels, it significantly enhances photo-degradation efficiency. The data suggest that optimal ammonia removal occurs at an H₂O₂ concentration of approximately 35 mM. In contrast, the phenol degradation model demonstrates that H_2_O_2_ plays a substantial role in improving degradation efficiency, with increased H_2_O_2_ dosage consistently contributing to enhanced phenol removal.

Figures 11j and 12j declares the interaction between Initial Ammonia concentration and phenol initial concentration. For ammonia degradation model, by increasing both ammonia and phenol concentration % removal of ammonia and phenol decreased. As Increasing the initial concentration of the target pollutant can reduce the overall efficiency of photocatalytic degradation. This decline is attributed to two primary factors: (1) higher substrate concentrations increase solution turbidity, which limits light penetration and reduces the number of photons reaching the photocatalyst surface; and (2) excessive pollutant levels can lead to the saturation of active sites on the photocatalyst, thereby diminishing photonic efficiency and potentially causing catalyst deactivation^61^.

#### Photocatalytic degradation of ammonia and phenol optimization

**Fig. 13:**
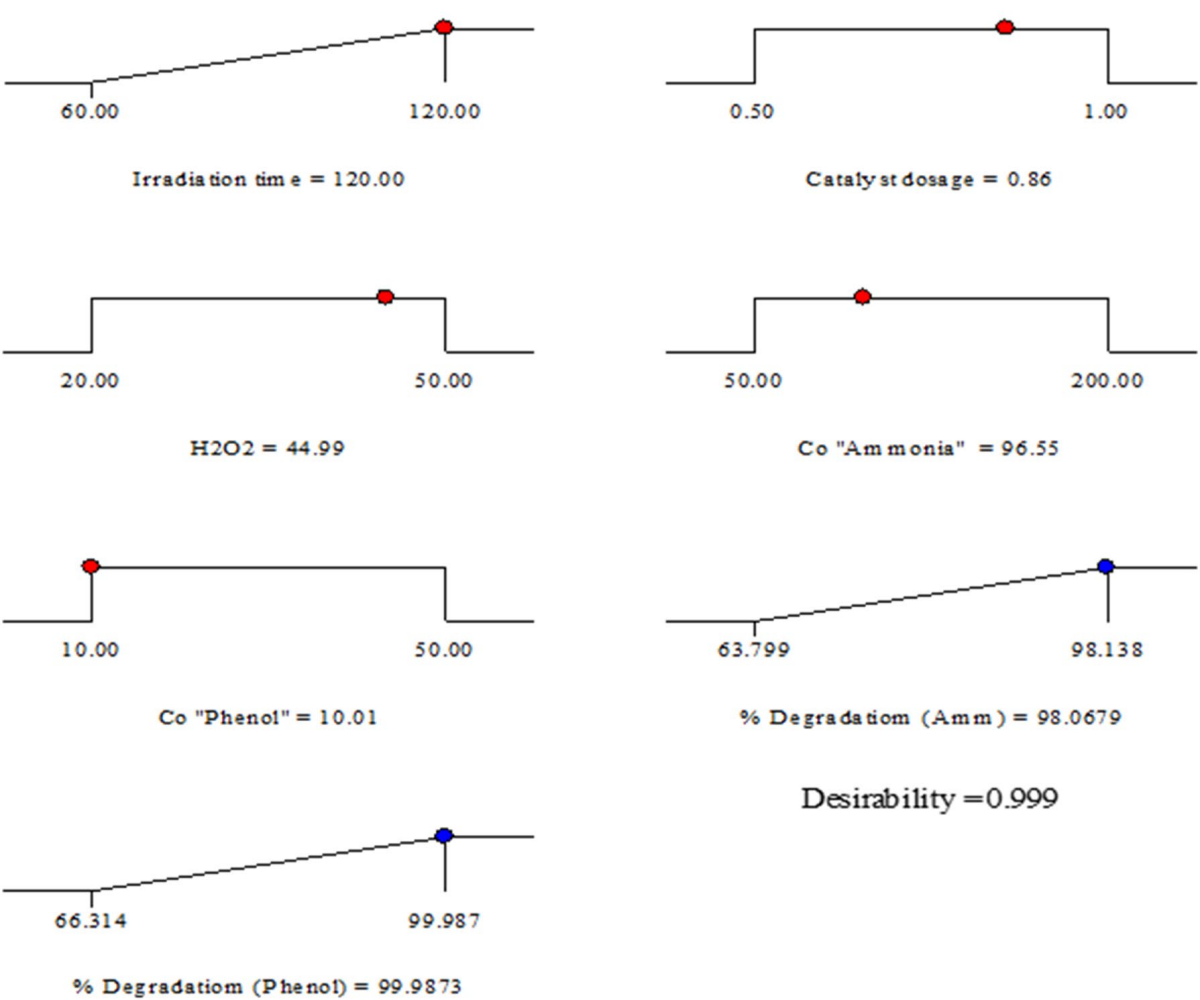
Desirability ramp for numerical optimization.

Optimization was performed using the desirability function to identify the most favorable conditions for the photocatalytic degradation of both ammonia and phenol. As the analysis aimed to maximize pollutant degradation, the operating parameters were adjusted to values within the examined range, while the response (photo-catalytic degradation efficiency for both ammonia and phenol) was adjusted to get a maximum value^64^. The optimal conditions, as shown in Fig. 13 were determined to be: irradiation time 120 min, catalyst dosage of 0.86 g L^-1^, H_2_O_2_ 45 mM, initial concentration of ammonia 96.55 ppm and initial concentration of phenol 10 ppm. At these conditions the maximum predicted value for Ammonia degradation is 98.0679% and for phenol is 99.9873% and the desirability factor is 0.999 which is close to 1. The experimental values for photo-catalytic degradation of ammonia and phenol at these conditions are 96.85% and 98.011% respectively which specifies the possibility and reliability of the model.

### Mechanism of photo-catalytic degradation activity for N-ZnO/AC and N-TiO_2_/AC catalysts

**Fig. 14:**
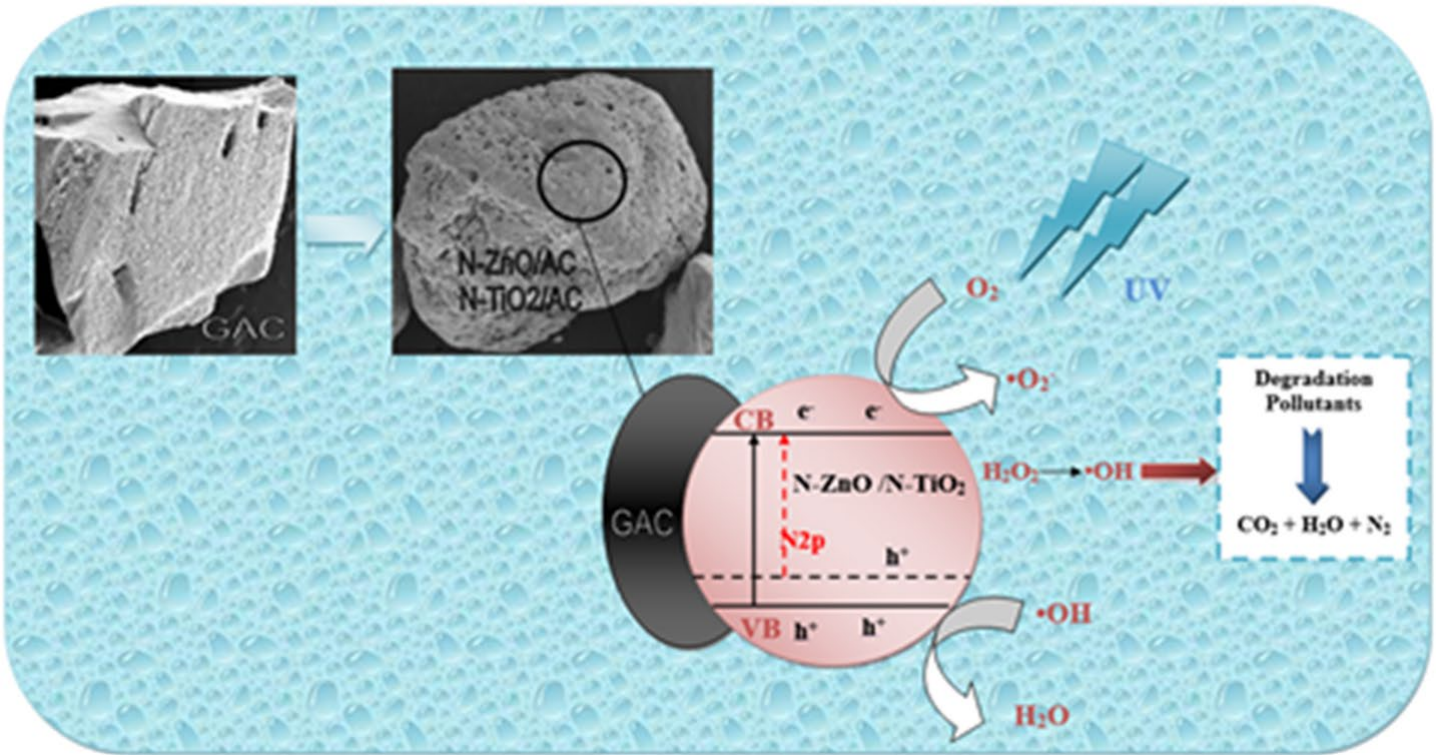
The mechanism of the photo-catalytic degradation of ammonia and phenol by N-ZnO/AC and N-TiO_2_/AC Catalysts.

Since GAC has particular morphological characteritcs that include its strong adsorption ability for several forms of organic and inorganic molecules, GAC is still the most regularly utilized adsorbent for air and water purification^65^. However, the absorption of light by loaded TiO2 and ZnO particles is limited, resulting in a reduction in the oxidation rate and the positive effects of GAC^43,65^. To address this issue as shown in Fig. 14, a composite system based on a unique structure was proposed, where the outer surface of GAC particles is fully covered with a porous TiO2 and ZnO film. Only the loaded TiO2 and ZnO can effectively absorb ultraviolet light^43,65^.

.

**Table 1:** The levels and ranges of variables in Box–Behnken statistical experiment design.

Independent variables	Symbol	Coded variable level		
		-1	0	1
Irradiation time “min”	A “X_1_”	60	90	120
Catalyst dosage “g/L”	B “X_2_”	0.5	0.75	1
H_2_O_2_ concentration “mM”	C“X_3_”	20	35	50
Co _Ammonia_ “mg/L”	D “X_4_”	50	125	200
Co _Phenol_ “mg/L”	E“X_5_”	10	30	50

**Table 2:** EDX analysis for catalysts.

Catalysts	Elements (Weight%)				
	O K	Zn K	Ti K	C K	*N* K
N-TiO_2_/ AC	20.67 ± 1.09		6.86 ± 0.32	64.56 ± 0.56	7.92 ± 1.06
N-ZnO/AC	22.98 ± 0.85	22.94 ± 1.09	-	45.18 ± 0.69	1.72 ± 0.49
ZnO	23.53 ± 0.24	76.47 ± 0.79	-	-	-
TiO_2_	46.08 ± 0.59	0.89 ± 0.10	30.77 ± 0.27	21.36 ± 0.16	-
GAC	7.31 ± 0.25			92.11 ± 0.22	

**Table 3:** Experimental matrix and results for RSM design.

A: Irradiation time “X_1_”	B: Catalyst dosage “X_2_”	C: H_2_O_2_ “X_3_”	D: Co “Ammonia” “X_4_”	E: Co “Phenol” “X_5_”	% Degradation - actual (Ammonia)	% Degradation - predicted (Ammonia)	% Degradation - actual (Phenol)	% Degradation - predicted (Phenol)
min	g/L	mM	mg/L	mg/L				
90	1	20	125	30	91.033	90.5704	90.553	90.4639
90	0.5	50	125	30	84.442	83.3133	86.378	85.7103
90	1	35	125	50	97.972	96.9691	94.906	95.0393
90	0.75	35	125	30	89.445	89.9736	90.305	90.8602
90	0.75	20	125	10	76.326	78.2371	87.053	87.958
60	0.75	35	125	10	78.061	77.4353	92.326	90.9877
90	0.75	35	200	50	83.915	83.1625	87.367	85.9333
90	0.5	35	50	30	85.093	84.5165	80.128	80.5186
120	0.75	35	200	30	92.379	91.0243	95.809	96.4204
90	0.75	35	200	10	85.059	84.0838	98.289	98.5367
120	0.75	50	125	30	93.12	95.3253	98.925	99.4898
120	0.75	35	50	30	97.838	95.882	97.275	96.9461
90	0.75	35	50	50	95.464	95.7438	89.957	88.8941
90	0.75	35	125	30	89.506	89.9736	90.353	90.6824
60	0.75	50	125	30	74.185	73.9974	82.296	82.1811
90	1	50	125	30	90.804	89.1514	95.599	95.6106
90	0.75	35	50	10	92.396	92.453	95.802	96.4204
90	0.75	35	125	30	88.994	88.9736	92.03	90.6824
90	0.75	20	125	50	80.817	82.7288	80.707	80.0951
90	0.75	50	125	50	88.867	88.4388	89.237	88.8717
90	0.75	20	200	30	77.468	75.5002	82.422	83.5674
120	0.75	20	125	30	83.042	83.8423	86.865	86.8312
90	0.75	35	125	30	90.912	89.9736	88.195	90.6824
90	0.75	50	125	10	90.99	89.521	99.987	101.139
90	0.5	35	125	50	81.246	80.5274	71.919	72.0401
60	0.75	20	125	30	69.039	67.4464	73.596	72.8824
60	0.75	35	200	30	65.405	66.5449	80.689	80.8951
90	0.5	35	125	10	79.215	79.5102	89.411	89.3719
90	0.75	20	50	30	81.002	80.3404	83.378	83.5442
90	1	35	200	30	89.723	91.3154	95.647	95.8286
60	0.75	35	50	30	82.099	82.6376	79.578	80.0289
90	0.5	20	125	30	63.799	63.8603	69.668	68.8997
90	0.5	35	200	30	67.235	69.3958	79.925	79.9519
90	0.75	50	200	30	78.725	78.8822	93.901	94.1006
120	0.5	35	125	30	87.355	86.6684	85.822	86.4304
60	0.75	35	125	50	76.708	76.8726	69.178	70.8778
90	1	35	125	10	95.606	95.6168	97.864	97.8372
120	1	35	125	30	98.138	98.828	98.241	98.0031
60	0.5	35	125	30	64.099	63.692	66.314	66.6421
90	0.75	35	125	30	91.011	89.9736	92.529	90.6824
120	0.75	35	125	10	94.794	94.5497	98.09	96.5714
120	0.75	35	125	50	96.936	97.4819	95.032	96.5516
90	1	35	50	30	96.29	96.1451	95.561	95.8021
90	0.75	50	50	30	93.529	94.9924	95.748	94.9634
60	1	35	125	30	83.111	84.0807	87.052	86.5339

**Table 4:** ANOVA results of the quadratic model for degradation of ammonia and phenol.

Source	Sum of squares	df	Mean square	F Value	*p*-value Prob > F	
Ammonia						
Model	3909.401	20	195.4701	92.79185	< 0.0001	Significant
A-Irradiation time “X_1_”	1423.081	1	1423.081	675.5528	< 0.0001	
B-Catalyst dosage “X_2_”	1059.389	1	1059.389	502.9038	< 0.0001	
C-H2O2 “X_3_”	325.2252	1	325.2252	154.3881	< 0.0001	
D-Co “Ammonia” “X_4_”	438.9235	1	438.9235	208.3619	< 0.0001	
E-Co “Phenol” “X_5_”	5.61453	1	5.61453	2.665281	0.1156	
AB - “X_1_ × _2_”	16.92911	1	16.92911	8.036441	0.0092	
AC “X_1_ × _3_”	6.081156	1	6.081156	2.886794	0.1022	
AD “X_1_ × _4_”	31.55631	1	31.55631	14.98014	0.0007	
AE “X_1_ × _5_”	3.053756	1	3.053756	1.449653	0.2403	
BC “X_2_ × _3_”	108.9101	1	108.9101	51.70086	< 0.0001	
BD “X_2_ × _4_”	31.87167	1	31.87167	15.12984	0.0007	
BE “X_2_ × _5_”	0.028056	1	0.028056	0.013319	0.9091	
CD “X_3_ × _4_”	31.75323	1	31.75323	15.07362	0.0007	
CE “X_3_ × _5_”	10.93625	1	10.93625	5.191561	0.0319	
DE “X_4_ × _5_”	4.435236	1	4.435236	2.105457	0.1597	
A^2 “X_1_^2^”	137.6717	1	137.6717	65.35433	< 0.0001	
B^2 “X_2_^2^”	52.59734	1	52.59734	24.96856	< 0.0001	
C^2 “X_3_^2^”	264.9816	1	264.9816	125.7898	< 0.0001	
D^2 “X_4_^2^”	27.47617	1	27.47617	13.04325	0.0014	
E^2 “X_5_^2^”	4.275436	1	4.275436	2.029598	0.1671	
Residual	50.55704	24	2.106543			
Lack of fit	47.14256	20	2.357128	2.761334	0.1674	Not significant
Pure error	3.414477	4	0.853619			
Cor total	3959.958	44				
*SD = 1.45*,* Mean = 85.18*,* R*^*2*^ *= 0.9872*,* Adjusted R*^*2*^ *= 0.9766*,* Predicted R*^*2*^ *= 0.9510*,* Adequate precision = 35.438 and CV% = 1.7.*						
Phenol						
Model	3317.408	20	165.8704	120.1957	< 0.0001	Significant
A-Irradiation time “X_1_”	977.0313	1	977.0313	707.992	< 0.0001	
B-Catalyst dosage “X_2_”	990.0148	1	990.0148	717.4003	< 0.0001	
C-H2O2 “X_3_”	482.1208	1	482.1208	349.3621	< 0.0001	
D-Co “Ammonia” “X_4_”	0.71318	1	0.71318	0.516796	0.4792	
E-Co “Phenol” “X_5_”	405.2068	1	405.2068	293.6275	< 0.0001	
AB - “X_1_ × _2_”	17.30144	1	17.30144	12.53725	0.0017	
AC “X_1_ × _3_”	2.8224	1	2.8224	2.045213	0.1656	
AD “X_1_ × _4_”	1.660232	1	1.660232	1.203064	0.2836	
AE “X_1_ × _5_”	100.902	1	100.902	73.11724	< 0.0001	
BC “X_2_ × _3_”	34.01222	1	34.01222	24.64648	< 0.0001	
BD “X_2_ × _4_”	0.02088	1	0.02088	0.015131	0.9031	
BE “X_2_ × _5_”	52.80929	1	52.80929	38.26751	< 0.0001	
CD “X_3_ × _4_”	0.19847	1	0.19847	0.143819	0.7078	
CE “X_3_ × _5_”	4.848804	1	4.848804	3.513618	0.0731	
DE “X_4_ × _5_”	6.443982	1	6.443982	4.669541	0.0409	
A^2 “X_1_^2^”	75.8061	1	75.8061	54.93182	< 0.0001	
B^2 “X_2_^2^”	84.74715	1	84.74715	61.41083	< 0.0001	
C^2 “X_3_^2^”	42.43177	1	42.43177	30.74758	< 0.0001	
D^2 “X_4_^2^”	3.400239	1	3.400239	2.463936	0.1296	
E^2 “X_5_^2^”	10.15501	1	10.15501	7.358684	0.0121	
Residual	33.12008	24	1.380003			
Lack of fit	21.45603	20	1.072801	0.3679	0.9411	Not significant
Pure error	11.66405	4	2.916013			
Cor total	3350.528	44				
*SD = 1.17*,* Mean = 88.26*,* R*^*2*^ *= 0.9901*,* Adjusted R*^*2*^ *= 0.9819*,* Predicted R*^*2*^ *= 0.9689*,* Adequate precision = 42.986 and CV% = 1.33.*						

The proposed photocatalytic mechanism for ammonia and phenol degradation using N-ZnO/AC and N-TiO2/AC catalysts is outlined as follows: The introduction of N^3−^ ions into the ZnO and TiO_2_ lattices leads to the production of an N-doped energy level higher than the O2p valence band. This doping results in narrowing of the band gap, and impurity levels are formed within the band gaps of N-ZnO/AC and N-TiO_2_/AC. Under UV illumination, electrons are easily moved from the valence band to the conduction band of the catalysts^18^. GAC excutes as an electron acceptor, facilitating the rapid migration of photoexcited electrons from the conduction bands of N-doped ZnO and N-doped TiO_2_ to the carbon layer^36,43^.

Under ultraviolet irradiation, electrons in the conduction band of the catalysts and carbon layer react with oxygen (O_2_) to form superoxide anion radicals (O_2_^·−^). The separation of holes (h+) leads to their reaction with water molecules (H_2_O), generating hydroxyl radicals (·OH)^43^. The adding of H_2_O_2_ to the photocatalytic process further enhances the production of ·OH radicals^50^. These radicals facilitate the degradation of ammonia and phenol over N-ZnO/AC and N-TiO_2_/AC composites as the following equations:

7 $${H_2}{O_2}~+h\upsilon ~ \to 2~\left( { \cdot OH} \right)$$

8 $$On~Valence~Band~:~~O{H^ - }~+~{h^+} \to ~ \cdot OH$$

9 $$On~Conduction~Band~:~~{O_2}+~{e^ - } \to ~O_{2}^{{ - \cdot }}$$

10 $$O_{2}^{{ - \cdot }}+{H_2}{O_2}~+{e^ - }~ \to O{H^ - }+ \cdot OH~$$

11

In case of ammonia Eq. (11) could be written as the following^54^:


12


The carbon layer serves as an electron collector and transporter, preventing the recombination of electron-hole pairs on the semiconductor particles^43^. The synergistic effect between the N-ZnO and N-TiO_2_ particles and the granular activated carbon layer contributes to the improved photo-catalytic performance under UV light irradiation^43^. The complete degradation of ammonia and phenol results in the formation of CO_2_, H_2_O, and N_2_ as degradation products^3,5,54,66^.

## Conclusions

The preparation of N-ZnO/AC and N-TiO_2_/AC as an effective photo-catalyst by using GAC as a support was achieved based on a simple procedure. The incorporation of nitrogen into N-ZnO/AC and N-TiO_2_/AC photo-catalysts was verified by applying different analysis such as EDX and XPS analyses. N-Doping causing a narrowing in band gap energy of N-ZnO/AC and N-TiO_2_/AC photo-catalysts which was revealed by DRS. The UV/catalyst/ H_2_O_2_ hybrid advanced oxidation process was more efficient than photolysis, photo-catalyst process and photolysis by UV irradiation and H_2_O_2_. Optimal degradation of ammonia and phenol was achieved at cyclic flow arte 8 L/min and moderate pH by using both N-ZnO/AC and N-TiO_2_/AC catalysts. RSM method was applied and N-ZnO/AC photo-catalyst activity was mainly affected by irradiation time, dose of catalyst, H_2_O_2_ concentration, initial ammonia concentration, and initial phenol concentration. Three-dimensional response surface plots were utilized to visualize the effects of these variables on the degradation efficiency of both pollutants. The regression coefficient of ammonia and phenol for experimental results indicated a strong concordance with the predicted values (R^2^ = 0.9872 and R^2^ = 0.9901, respectively). At the optimal conditions of 120 min irradiation time, 0.86 g L^− 1^ catalyst dose, 45 mM H_2_O_2_, 96.6 mg/L and 10 mg/L ammonia and phenol initial concentration, respectively, the degradation efficiency of ammonia and phenol approached 96.9% and 98.0%, respectively. Under UV irradiation, the N-ZnO/AC catalyst exhibited particularly high photocatalytic performance in the degradation of both pollutants.

## Data Availability

The datasets generated and/or analysed during the current study are available from the corresponding author on reasonable request.
